# RNA modifications stabilize the tertiary structure of tRNA^fMet^ by locally increasing conformational dynamics

**DOI:** 10.1093/nar/gkac040

**Published:** 2022-02-07

**Authors:** Thomas Biedenbänder, Vanessa de Jesus, Martina Schmidt-Dengler, Mark Helm, Björn Corzilius, Boris Fürtig

**Affiliations:** Institute for Organic Chemistry and Chemical Biology, Center for Biomolecular Magnetic Resonance (BMRZ), Johann Wolfgang Goethe-Universität, Frankfurt am Main 60438, Germany; Institute of Chemistry and Department Life, Light & Matter, University of Rostock, Rostock 18059, Germany; Institute for Organic Chemistry and Chemical Biology, Center for Biomolecular Magnetic Resonance (BMRZ), Johann Wolfgang Goethe-Universität, Frankfurt am Main 60438, Germany; Institut für pharmazeutische und biomedizinische Wissenschaften (IPBW), Johannes Gutenberg-Universität, Mainz 55128, Germany; Institut für pharmazeutische und biomedizinische Wissenschaften (IPBW), Johannes Gutenberg-Universität, Mainz 55128, Germany; Institute of Chemistry and Department Life, Light & Matter, University of Rostock, Rostock 18059, Germany; Institute for Organic Chemistry and Chemical Biology, Center for Biomolecular Magnetic Resonance (BMRZ), Johann Wolfgang Goethe-Universität, Frankfurt am Main 60438, Germany

## Abstract

A plethora of modified nucleotides extends the chemical and conformational space for natural occurring RNAs. tRNAs constitute the class of RNAs with the highest modification rate. The extensive modification modulates their overall stability, the fidelity and efficiency of translation. However, the impact of nucleotide modifications on the local structural dynamics is not well characterized. Here we show that the incorporation of the modified nucleotides in tRNA^fMet^ from *Escherichia coli* leads to an increase in the local conformational dynamics, ultimately resulting in the stabilization of the overall tertiary structure. Through analysis of the local dynamics by NMR spectroscopic methods we find that, although the overall thermal stability of the tRNA is higher for the modified molecule, the conformational fluctuations on the local level are increased in comparison to an unmodified tRNA. In consequence, the melting of individual base pairs in the unmodified tRNA is determined by high entropic penalties compared to the modified. Further, we find that the modifications lead to a stabilization of long-range interactions harmonizing the stability of the tRNA’s secondary and tertiary structure. Our results demonstrate that the increase in chemical space through introduction of modifications enables the population of otherwise inaccessible conformational substates.

## INTRODUCTION

tRNAs show the highest modification rates of all known RNAs. Among the over 170 modified nucleotides known so far at least 123 occur in tRNAs (1–5). Remarkably, in *Escherichia coli* one percent of the genome codes for proteins involved in post-transcriptional nucleotide modification. This highlights the functional impact of the increased chemical diversity on tRNA biology (6–13).

However, irrespective of the number and kind of modifications and of the vast different primary structures (14), all tRNAs adopt the same secondary (cloverleaf) and highly folded tertiary structure (L-shape). This iconic three dimensional structure was first shown for yeast tRNA^Phe^ (15). It is virtually identical for all tRNAs across the biological kingdom. As common for all tRNAs, its secondary cloverleaf structure is divided into five parts, the acceptor stem (Acc. stem), the dihydrouridine arm (D-arm), the anticodon stem loop (ACSL), a variable loop and the TΨC-arm. The typical L-shaped tertiary structure is mainly formed by a plethora of interactions between the D-arm and TΨC-arm.

Modifications within tRNA are classified by their functional role (16). Modifications in the anticodon stem loop (ACSL) modulate the cognate codon-anticodon interaction and therefore have a regulatory function during decoding (17,18). In contrast, modifications in the core of the tRNA influence the tertiary fold and thus are mainly found at the interface between D- and TΨC-arm. They maintain the balance between flexibility and stability within the L-shaped fold (19). The structural impact of modifications depends on their type and position within the tRNA. It ranges from influencing the hydrophobic character of a base, its stacking ability and base pairing properties up to the net charge of the nucleotide (reviewed in (20)).

Among all tRNAs, tRNA^fMet^ possesses not only a unique function but also at least three unique structural features. In bacteria, it initiates translation by decoding the start codon AUG of the mRNA. tRNA^fMet^ directly binds to the P-site of the ribosome, whereas elongator-tRNAs first bind to the ribosomal A-site and then translocate to the P-site. The ability of initiator tRNA^fMet^ to bind directly to the P-site is attributed to the formylated methionine. Structurally, tRNA^fMet^ contains three uniquely conserved GC base pairs (21,22) in the ACSL (G29-C41, G30-C40 and G31-C39). A C1-A72 mismatch functions as recognition site for the formylation (23). The base pair A11-U24 represents a purine-pyrimidine pair, whereas all other tRNA contain a pyrimidine-purine base pair at this position (23). In addition, the structure contains the wobble-like C_m_32:A38 base pair (24). The atomic details of the structure were solved by X-ray crystallography (25) and the most recent structure exhibits a resolution of 3.1 Å (24). Besides the C_m_32 modification in the ACSL, four core modifications were found: 4-thiouridine 8 (s^4^U8), dihydrouridine 20 (D20), ribothymidine 54 (T54) and pseudouridine 55 (Ψ55). All the core modifications except dihydrouridine are generally assumed to thermally stabilize the tRNA structure due to the higher structural rigidity of the nucleosides introduced by these modifications (20,26).

A number of studies have focused on the role of singular modifications regarding structure or function (19,27–29). For example, it could be shown that T54 has a high impact on the thermal stability of elongator tRNA^Met^ (29). Thiolation of U8 changes base pairing properties, functions as UV protective in tRNAs and stabilizes tRNA structure (20,30,31). The thio-modification at position U8 is also highly conserved in bacteria.

Exhibiting a non-aromatic and non-planar base, dihydrouridine cannot contribute to stacking interactions in helices and thus is supposed to increase the flexibility of the D-arm (19,27). Pseudouridine (Ψ) being one of the most abundant base modification, has, due to its C–C sugar bond, an additional imino group involved in hydrogen bonding, e.g. with localized water molecules (27,32). Moreover, it can base pair with all four canonical bases. The Ψ modification can be found over the whole tRNA primary structure, but Ψ55 is highly conserved. However, little is known about the impact of the entirety of modifications on the local and global dynamics of a tRNA molecule (33). It has been hypothesized that the modifications lead to a stabilization of tRNA structure by simultaneously maintaining its flexibility needed for their proper biological function. Comparative structural analyses revealed that the overall structures of tRNAs are identical (34,35), although modifications can increase the surface area of tRNA up to 20% (26). Interestingly, unmodified tRNAs can adopt several structural conformations (34,36–42), highlighting the fact that in several cases the modifications are needed to restrict the conformational space. Along this line, it could be shown that *in vitro* in absence or at low concentrations (<2 mM) of Mg^2+^, the tertiary fold of non-modified are less stable than modified tRNAs (35,43). Further, some results hint at a variation in the local stabilities that influence the structural behavior of the tRNAs at saturating (44) (>5 mM) concentrations of Mg^2+^. Recently, the structural comparison between the crystal structure of *E. coli* tRNA^Phe^ and the yeast analogue revealed a different angle between the acceptor and anticodon stems as well as a different arrangement of a triplet base pair (42). On the other hand, experiments investigating differences between non-modified and modified *E. coli* tRNA^Val^ showed that modifications do not alter the global structure (34). It is important to note, that the concentration of Mg^2+^ critically influences the globular arrangement of the tRNA and under non-saturating concentrations globular structural differences might occur between modified and non-modified tRNAs (45). Investigation of local base pair dynamics were performed by deuterium exchange rates at 5°C showing a difference in dynamics between non-modified and modified tRNA. However, detailed investigations about local base pair dynamics are still missing.

Here, we investigate the impact of modifications on the structural dynamics of *E. coli* tRNA^fMet^ at single nucleotide resolution by nuclear magnetic resonance (NMR) spectroscopy. We performed a comprehensive analysis between modified and non-modified tRNAs on different time scales to evaluate the local and global dynamics. We further investigated the global stability and structural plasticity of both tRNAs in comparison. As reported before, we find that the introduction of modifications does not alter the global structure of tRNA by means of nucleotide interactions responsible for the formation of secondary and tertiary interactions (34–44). However, our data clearly shows that the introduction of nucleotide modifications preserves or even enhances local dynamics. This affects also long-range interactions of nucleobases that get stabilized over a wide temperature range. Although the modifications are silent with respect to the overall structure, in consequence of these two effects the thermodynamic stability of tRNA^fMet^’s secondary and tertiary structure gets harmonized. Through this harmonization the two seemingly opposing properties of having a structural stable but at the same time flexible tRNA molecule are enabled. Our results lead to new insights for understanding how the modifications interfere with and modulate local dynamics and are correlated to the global stability of tRNAs.

## MATERIALS AND METHODS

### Preparation of unmodified tRNA^fMet^

The DNA template of 77 nt tRNA^fMet^ from *E. coli* is flanked by two restriction sites and a hammerhead ribozyme: (transcribed sequence underlined) GAATTC (EcoRI) – TAATACGACTCACTATAG (T7 promotor) – GGACCCCGCGCTGATGAGTCCGTGAGGACGAAAGACCGTCTTCGGACGGTCTC (hammerhead ribozyme) – CGCGGGGUGGAGCAGCCUGGUAGCUCGUCGGGCUCAUAACCCGAAGAUCGUCGGUUCAAAUCCGGCCCCCGCAACCA (tRNA^fMet^) – TATG (NdeI).

Plasmid DNA was purchased from Genscript (New Jersey, USA). DNA was amplified by polymerase chain reaction (PCR) using T7 forward primer (5′-TAATACGACTCACTATAGG-3′) and tRNA reverse primer (5′-TGGTTGCGGGGGCC-3′). Primers were purchased from Eurofins MWG Operon (Ebersberg, Germany).

^13^C–^15^N-labeled 77 nt tRNA^fMet^ was synthesized by *in vitro* transcription with T7 RNA polymerase from PCR product as described in literature (46,47). The ^13^C–^15^N-labeled (rATP, rCTP, rGTP, rUTP) nucleotides for transcription were purchased from Silantes (Munich, Germany). The construct was purified by preparative polyacrylamide gel electrophoresis according to standard protocols (48). The construct was folded in water for 5 min at 95°C and immediately diluted 10-fold with ice-cold water. The RNA was buffer-exchanged into NMR buffer (25 mM potassium phosphate, 200 mM KCl, 5 mM MgCl_2_, pH 6.2).

### Preparation of modified tRNA^fMet^

The plasmid DNA template (pBStRNAfmetY2) (49) was kindly provided by Emanuelle Schmitt. The gene of interest is flanked by a *lpp* promoter, a 5′-flanking sequence, and a *rrnC* terminator sequence (50–52). The plasmid was transformed into competent JM101tr cells.

For expression of native tRNA^fMet^, pre-culture was grown in 5 ml LB medium at 37°C for 4 h. Afterwards, the culture was centrifuged at 4000 g for 5 min and the supernatant was discarded. The pellet was resuspended in 100 ml M9 medium, which contained uniformly labeled ^13^C-glucose and ^15^N-ammonium chloride as the sole carbon and nitrogen supplies. Cell cultures were grown at 37°C overnight. On the next day, the culture was transferred to 2 l of uniformly labeled ^13^C,^15^N-M9 medium for further expression. The culture was incubated at 37°C for 24 h, the culture was harvested, and the supernatant was discarded.

The cell pellet obtained from the expression was resuspended in 10 ml of buffer T1 (1 mM Tris–HCl, pH 7.4, 10 mM MgCl_2_). The suspension was mixed with one equivalent of T1-saturated phenol. Thereafter, the mixture was vortexed for 3 min and then slowly shaken for 1 h. After incubation, the preparation was centrifuged at 10 000 g and RT for 1 h to separate the phenol and the water phase. The water phase was carefully recovered, whereas the phenol phase was mixed again with 2 mL of buffer T1 for counter-extraction. The total soluble phase, which contains total nucleic acids from bacteria, was mixed with 1 volume equivalent chloroform to remove remaining phenol. Again, phase separation was achieved by centrifugation at 10 000 g and 25°C for 20 min. After recovering of the soluble phase, the preparation was mixed with 5 M NaCl solution to reach a final concentration of 0.5 M and then mixed with 2.5 volume equivalents of ice-cold ethanol. Before centrifuging the mixture at 10 000 g and 4°C for 1 h, the mixture was vortexed for 10 s. The supernatant was discarded, and the pellet was resuspended in 5 ml of 1 M sodium chloride. The preparation was again centrifuged at 10 000 g and 4°C for 1 h. Ice-cold ethanol (2.5 volume equivalents) was added to the supernatant and centrifuged again at 10 000 g and 4°C for 1 h. The supernatant was discarded, and the pellet was resuspended in 2 ml of 1.8 M Tris–HCl buffer (pH 8.0). Afterwards, the preparation was incubated at 37°C for 2 h to insure a full deacylation of all tRNA molecules. The preparation, which contains total tRNA extract without any aminoacylation, was mixed with 0.2 ml 5 M NaCl and 2.5 volume equivalents ice-cold ethanol for precipitation. After centrifuging, the preparation at 10 000 g and 4°C for 1 h, the pellet was resuspended in 4 ml of T2 buffer (20 mM Tris–HCl, pH 7.5, 0,1 mM EDTA, 0.2 M NaCl) for further purification by anion exchange chromatography.

Anion exchange chromatography was performed to purify the native tRNA^fMet^ construct. The corresponding protocol was modified (49). For this, a HiPrep Q Sepharose Fast-Flow column (GE Healthcare) was connected to an Äkta^®^ purifier system (GE Healthcare) and RNA of various concentration was injected. The flow rate was set to 5 ml/min. Unbound RNA fragments were eluted with one column volume (CV, 53 ml) at a sodium chloride concentration of 0.48 M. A gradient was applied from 0.48 to 0.52 M sodium chloride. The change of salt concentration per hour was 0.06 M/h. An isocratic flow step at 0.52 M was then applied for either 5 CV, 6 CV or 8 CV. A second gradient (0.06 M/h) was applied from 0.516 to 0.544 M sodium chloride followed by a further, but steeper gradient (2 M/h) from 0.544 to 1 M to elute all remaining RNA. Eluate was collected and analyzed with denaturing PAGE.

### Liquid chromatography-tandem mass spectrometry (LC-MS/MS)

150 ng of total RNA per sample was digested to nucleosides using 0.6 U nuclease P1 from *P. citrinum* (Sigma-Aldrich), 0.2 U snake venom phosphodiesterase from *C. adamanteus* (Worthington), 0.2 U bovine intestine phosphatase (Sigma-Aldrich), 10 U benzonase (Sigma-Aldrich), 200 ng Pentostatin (Sigma-Aldrich) and optional 500 ng tetrahydrouridine (Merck-Millipore) in 5 mM Tris (pH 8) and 1 mM magnesium chloride for two hours at 37°C. 30 ng of digested RNA was mixed with 25 ng of internal standard (^13^C stable isotope-labeled nucleosides from *E. coli*) and subjected to LC–MS analysis. Nucleoside levels were measured using an Agilent 1260 Infinity system in combination with an Agilent 6470 Triple Quadrupole mass spectrometer equipped with an electrospray ion source (ESI)). The solvents consisted of 5 mM ammonium acetate buffer (pH 5.3, adjusted with acetic acid; solvent A) and LC–MS grade acetonitrile (solvent B; Honeywell). A C18 reverse HPLC column (Synergi™ 4 μM particle size, 80 Å pore size, 250 × 2.0 mm; Phenomenex) was used at a temperature of 35°C and a constant flow rate of 0.35 ml/min was applied. The compounds were eluted with a linear gradient of 0–8% solvent B over 10 min, followed by 8–40% solvent B over 10 min. Initial conditions were regenerated with 100% solvent A for 10 min. The four main nucleosides were detected photometrically at 254 nm via a diode array detector (DAD). The following ESI parameters were used: gas temperature 300°C, gas flow 7 l/min, nebulizer pressure 60 psi, sheath gas temperature 400°C, sheath gas flow 12 l/min, capillary voltage 3000 V, nozzle voltage 0 V. The MS was operated in the positive ion mode using Agilent MassHunter software in the dynamic MRM (multiple reaction monitoring) mode. For absolute quantification, internal and external calibration was applied as described in Thüring *et al.* (53). The total amount of modified nucleosides was normalized to the amount of injected RNA molecules.

### Circular dichroism (CD)

For the determination of the melting behavior of the tRNA^fMet^, CD melting profiles were measured. One sample from each construct was therefore prepared with an absorption at 260 nm of ∼1–2 AU.

All CD spectra and melting curves were acquired on a JASCO J-810 spectropolarimeter with constant influx of nitrogen to prevent formation of ozone. The spectra were acquired in a quartz cuvette with 1 mm path length with a sample volume of 200 μl. Melting curves were acquired at constant wavelength (265 nm), where largest change in molar ellipticity upon heat denaturation was expected, typically using a temperature ramp rate of 0.5°C/min.

The melting curves were evaluated according to the method of Marky and Breslauer (54). In short, two baselines representing the maximal and minimal value of CD ellipticity were determined. The percentage of folded RNA was then calculated by normalizing the measured CD ellipticity as described. Percentage of folded RNA was then non-linear regressed against the temperature with the following equation:

$$\begin{equation*}\alpha \ = \ a + \left( {b - a} \right) \times \frac{{\exp \left( { - \frac{{\Delta H - T\Delta S}}{{RT}}} \right)}}{{1 - \exp \left( { - \frac{{\Delta H - T\Delta S}}{{RT}}} \right)}}\end{equation*}$$

### NMR spectroscopy

All experiments were performed at 25°C in NMR buffer (25 mM potassium phosphate, 200 mM KCl, 5 mM MgCl_2_, pH 6.2) and 5–10% D_2_O. All spectra were referenced to DSS (4,4-dimethyl-4-silapentane-1-sulfonic acid). Nitrogen-15 and Carbon-13 chemical shifts were indirectly referenced using the ratio of the gyromagnetic ratios of proton to ^15^N (0.101329118) and ^13^C (0.251449530), respectively (55). NMR experiments were performed on 700, 800, 900 or 950 MHz NMR spectrometer equipped with a 5 mm, *z*-axis gradient ^1^H {^13^C, ^15^N} TCI cryogenic probe. NMR Experiments were analyzed using Bruker Biospin software TopSpin 3.5. Assignments were performed using the software Sparky 3.114 (56).

### 2D-[^1^H,^15^N]-BEST-TROSY and 2D-[^1^H,^15^N]-HSQC experiments

BEST-TROSY (Band-selective excitation short-transient – transverse relaxation-optimized spectroscopy) and HSQC (Heteronuclear single quantum coherence) experiments were performed to observe ^15^N–^1^H correlations of uniformly ^13^C,^15^N-isotope labeled tRNA^fMet^ (57–59). Moreover, signal of imino protons can only be detected for nucleotides in stable base pairs, which protects the imino proton from fast solvent exchange (46). However, the ‘imino’ proton of the 5,6-dihydrouridine (D) can be observed even if the nucleoside is not part of a stable base pair due to its higher p*K*_a_ (19). For BEST-TROSY experiments, the pulse program with the modification proposed by Brutscher and coworkers was used (58,59). For each experiment, the used parameters are listed in the respective figure caption.

### 2D-[^1^H,^15^N]-HNN-COSY and 3D-[^1^H,^15^N,^15^N]-HNN-COSY experiments

The HNN-COSY experiment correlates a NH group with a hydrogen-bonded N-atom by a hydrogen bond scalar coupling (HBC) (60). 2D-[^1^H,^15^N]-HNN-COSY and 3D-[^1^H,^15^N,^15^N]-HNN-COSY experiments were acquired with 45 ms and 30 ms ^15^N–^15^N transfer delay, respectively.

### 2D-[^1^H,^1^H]-NOESY and 3D-[H^1^H,^15^N,^1^H]-SOFAST-HMQC-NOESY experiments

2D-[^1^H,^1^H]-NOESY and 3D-[^1^H,^15^N,^1^H]-SOFAST-HMQC-NOESY experiments were acquired using a pulse program with jump-return water suppression (2D-NOESY) or Watergate water suppression (3D-NOESY) and a carrier frequency switch between the direct and indirect dimension. For the direct dimension, the proton carrier frequency was set to the water resonance frequency (4.7 ppm), whereas it was switched to either 8.85 ppm or 7.29 ppm in the indirect dimension. The nitrogen carrier frequency was set to 153.25 ppm. All spectra were recorded with a spectral width of 25 ppm in the direct dimension, 16 ppm in the indirect proton dimension and to either 19.2 ppm or 22.5 ppm in the indirect nitrogen dimension. The mixing time for the through-space coherence transfer was set to 150 ms.

### Sofast-^1^H,^31^P HMQC experiments

For the HP-correlation across hydrogen bond a sofast-HMQC experiment (61) was conducted on a Bruker AVIII 700MHz spectrometer equipped with a cryogenic-QCI HCNP probe. The transfer timer was set to 16.6ms, and pulses were applied at frequencies of 12.5, 0 and 135 ppm for ^1^H, ^31^P and ^15^N, respectively. Selective proton pulses were applied with a bandwidth of 4.8 ppm. Hard ^31^P pulses were applied with a bandwidth of 5.81 kHz and ^15^N decoupling with a bandwidth of 1 kHz. The recycling delay was set to 0.5 s.

### Determination of ^15^N relaxation parameters

^15^N relaxation parameters were measured as described (62,63) and were determined with the program DynamicsCenter, version 2.6.2 (Bruker Biospin). A delay of either 1.5 s at a magnetic field of 600 MHZ or 2.8 s at a magnetic field of 800 MHz was used as relaxation delay. For determination of the ^15^N spin-lattice relaxation rate *R*_1_ up to 20 mixing times between 20 ms and 2.8 s were applied. In case of the ^15^N spin-spin relaxation rate, multiples of the 16.96 ms CPMG length of up to 12 were applied. For an accurate error estimation in all measurements, two mixing times were repeated.

In short, the three parameters comprising of the spin-lattice relaxation rate *R*_1_, the spin-spin relaxation rate *R*_2_, and the steady-state heteronuclear *NOE* (*hetNOE*) were measured by a series of 2D-[^1^H,^15^N]-HSQC experiments as described in the following.

Determination of ^15^N-spin-lattice relaxation rates (*R*_1_) were conducted by measuring a series of inversion-recovery experiments with varying recovery delays ${\tau _M}$ as a pseudo-3D-[^1^H,^15^N]-HSQC-${R_1}$ experiment. Fitting of the time-modulated signal intensities with the following equation yields the ^15^N-spin-lattice relaxation rate:

$$\begin{equation*}I\ \left( t \right) = {I_0}{\rm{\ }} \cdot \exp \left( { - {R_1} \cdot {\tau _M}} \right)\end{equation*}$$

The ^15^N-spin-spin relaxation rate (*R*_2_) describes the decay of the transverse magnetization by loss of phase coherence, which is mainly driven by dipolar interaction with adjacent spins. The obtained signal intensity is plotted against the recovery delays and fitted with a mono-exponential function resulting in the ^15^N-*R*_2_ value:

$$\begin{equation*}I\ \left( t \right) = {I_0}{\rm{\ }} \cdot \exp ( - {R_2} \cdot {\tau _M})\end{equation*}$$

The $hetnOe$ is a cross-relaxation phenomenon(64) and thus can be used to calculate the ^1^H-^15^N cross relaxation (63). The $hetnOe$ of each imino group was determined by either an interleaved 2D-[^1^H,^15^N]-HSQC-$hetNOE$ at 600 MHz or a pseudo-3D-[^1^H,^15^N]-HSQC-$hetNOE$ at a field strength of 800 MHz. Both experiments measure one set of data with prior proton saturation and another set without proton saturation. The $hetNOE$ value is extracted from both sets by calculating the quotient of saturated and unsaturated signal intensity:

$$\begin{equation*}{\rm{\ }}\frac{{{I^{saturated}}}}{{{I^{unsaturated}}}} = \ 1 + \frac{{{\gamma _H}{\sigma _{NH}}}}{{{\gamma _N}{R_1}}}\end{equation*}$$

### Model-free analysis

The determined relaxation parameters were used as input for model-free analysis (65,66) with the program relax, version (4.1.3) (67,68) using a virtual machine provided by NMRBox (69). Analysis was performed at two temperatures (298 and 313 K) with the imino bond length and imino chemical shift anisotropy determined by Grishaev *et al.* (70). Parameters of the motion models as described in the relax manual were fit to the data. Thereby, the diffusion seed paradigm was used as described at 298 K (71). The resulting diffusion tensor was fixed for the Model-free analysis at 313 K, the global rotational correlation time and the $\frac{{{{\rm{D}}_\parallel }}}{{{{\rm{D}}_ \bot }}}$ ratio was calculated as described by Fushman (72). The Model-free selection was performed using the Akaike's Information Criterion (AIC) (73).

### Measurement of selective water inversion recovery experiments

The solvent exchange rates of imino protons were measured as previously described (74–76). The resulting pseudo-2D-[^1^H,${\tau _M}$]-jump-return experiments were measured at six different temperatures from 10°C to 55°C. In total, 18 recovery delays were applied in a range from 1 ms to 3200 ms. The buffer contained 25 mM KPi (pH 6.2), 200 mM KCl and 5 mM MgCl_2_. The experiment was recorded with 40 scans and 18 delays for *τ_M_*. Carrier frequencies were set to 4.697 ppm (direct dimension) and 153.5 ppm (15N). The spectral width was set to 25 ppm. Radiofrequency field strength of the proton hard pulse were set to 13.47 kHz. The hard pulse for ^15^N decoupling was set to 6.41 kHz. The relaxation delay was set to 5 s and the acquisition time was set to 51.2 ms. Garp4 was used as a decoupling scheme during acquisition. Analysis of the imino exchange rate was applied according to the proposed theory (74–76). ${\rm{\Delta \Delta }}{G_{diss}}$ values were calculated by subtracting the individual ${\rm{\Delta }}{G_{diss}}$ values of the non-modified tRNA from the modified tRNA values.

## RESULTS

Modified tRNA^fMet^ was obtained by overexpression in ^13^C–^15^N-enriched minimal medium followed by a non-denaturing purification mainly through ion-exchange chromatography. High amounts (1.6 mg/l) of pure labeled tRNA^fMet^ suitable for NMR experiments were achieved (Supplementary Figure S1).

**Figure 1.**
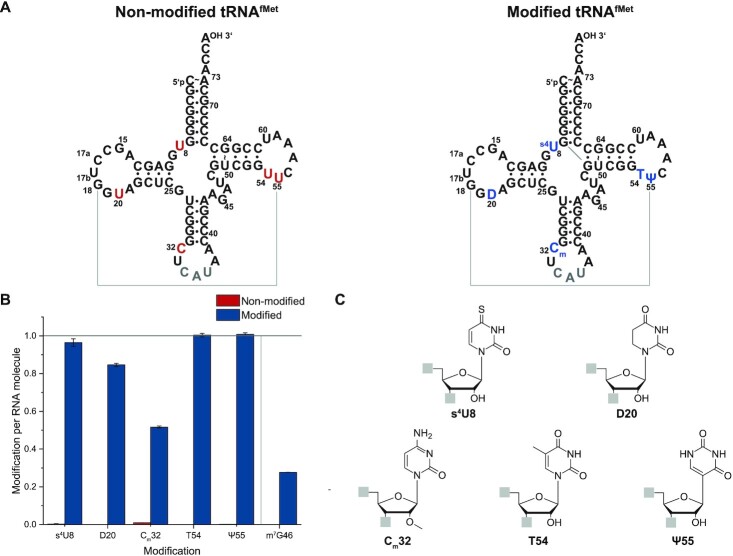
Quantification of modified nucleosides levels in the non-modified (red) and modified (blue) tRNA^fMet^ constructs. (**A**) Secondary structure of non-modified and modified tRNA^fMet^. (**B**) LC–MS based quantification of modified nucleosides levels in tRNA^fMet^. Both tRNA^fMet^ constructs were digested to nucleosides and analyzed via LC-MS. The amount of each modification was normalized to the amount of injected RNA molecules. The occurrence of one modified nucleoside per RNA molecule is highlighted with a black line. The modified nucleosides are s^4^U8 (thiouridine 8), D20 (5,6-dihydrouridine 20), C_m_32 (2′O-methylcytidine 32), T54 (ribothymidine 54), Ψ55 (pseudouridine 55), and m^7^G46 (7-methylguanosine 46). The measured relative abundance for m^7^G46 is based on the endogenous isoacceptor of tRNA^fMet^ in *E. coli*. In *E. coli* K strains, the initiator tRNA^fMet^ is encoded by four genes that differ by a single nucleotide at position 46 resulting in two tRNA^fMet^ species (79,80): metZ, metW and metV encode tRNA^fMet1^ with m^7^G46 and metY encode tRNA^fMet2^ with A46. Therefore, the cellular tRNA^fMet^ pool consists of 75% tRNA_G46_^fMet1^, and 25% of tRNA_A46_^fMet2^ due to the presence of three genes encoding for m^7^G46 and only one gene encoding for A46 (80,81). In contrast, the results here show only a relative abundance of 27.7% and thus, the second isoacceptor reflecting the tRNA_A46_^fMet2^ of interest is successfully overexpressed. (**C**) Chemical structure of analyzed modified nucleosides.

The degree of modification was evaluated by liquid chromatography-tandem mass spectrometry (LC-MS/MS). All known modifications present in *E. coli*’s tRNA^fMet^ were detected. For the four modified nucleotides within the core of tRNA^fMet^, degrees of modification between 85 ± 1% for D20 and 101 ± 1% for Ψ55 are found (Figure 1 and Supplementary Figure S2).

The methoxy modification of C_m_32 shows only a relative abundance of 52% ± 1% and thus representing a hypomodification of C_m_32 in our sample. In general, it was shown that the degree of modification varies with growth and stress conditions, e.g. growth temperature or starvation (77,78). However, it was recently shown that the C_m_32 modification of initiator tRNA^fMet^ in *E. coli* is not affected by different growth conditions and temperatures (77).

The measured relative abundance of 27.7% ± 0.1% for m^7^G46 is based on the endogenous isoacceptor of tRNA^fMet^ in *E. coli*. In *E. coli* K strains, the initiator tRNA^fMet^ is encoded by four genes resulting in two tRNA^fMet^ species (79): metZ, metW and metV encode tRNA^fMet1^ and metY encode tRNA^fMet2^. The tRNAs differ by a single nucleotide at position 46, where tRNA^fMet1^ contains an m^7^G46 and tRNA^fMet2^ an A46 (80). Therefore, the cellular tRNA^fMet^ pool consists of 75% tRNA_G46_^fMet^, and 25% of tRNA_A46_^fMet^ due to the presence of three genes encoding for m^7^G46 and only one gene encoding for A46 (80,81). In contrast, the results here show only a relative abundance of 27.7% and thus, the second isoacceptor reflecting the tRNA^fMet^ of interest is successfully overexpressed.

**Figure 2.**
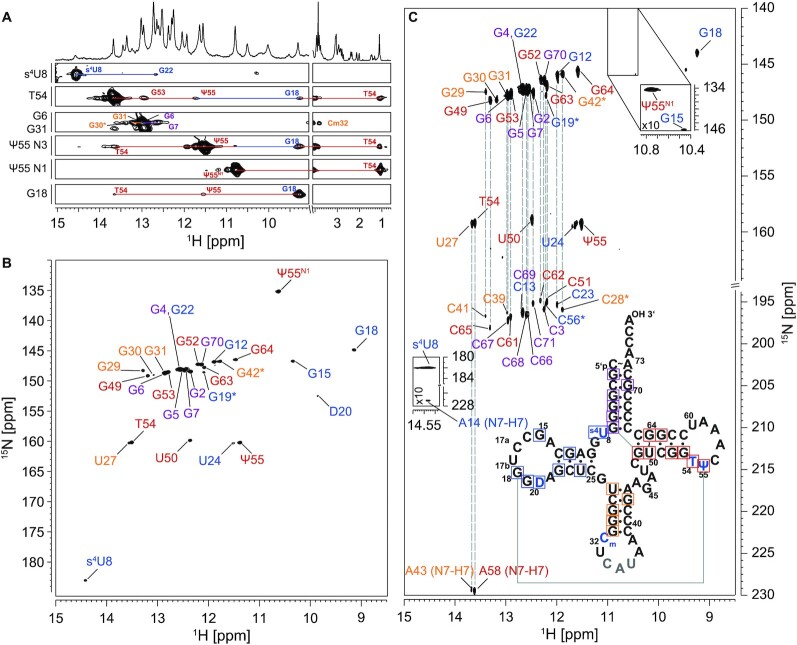
Selected [^1^H,^1^H]-strips of a 3D-[^1^H,^15^N,^1^H]-SOFAST-HMQC-NOESY (**A**), 2D-[^1^H,^15^N]-BEST-TROSY (**B**) and 2D-[^1^H,^15^N]-BEST-TROSY-HNN-COSY experiment (**C**) of modified tRNA^fMet^ at 25°C. Secondary structure of the modified tRNA^fMet^ is shown in (C). Unambiguously assigned imino peaks are highlighted in either purple (acceptor stem), blue (D-arm), yellow (ACSL), or red (TΨC-arm). Experimental details are provided in an extended figure caption in SI.

**Figure 3.**
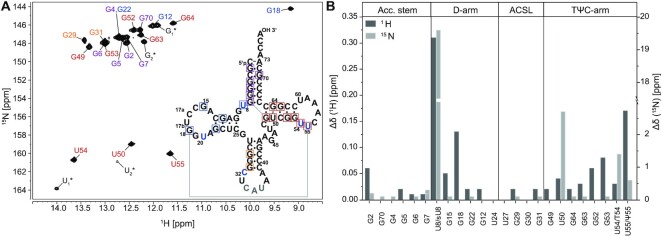
2D-[^1^H,^15^N]-BEST-TROSY experiment of unmodified tRNA^fMet^ with secondary structure (**A**) and analysis of chemical shift differences between modified and non-modified tRNA (B). (A) The BEST-TROSY spectrum of the non-modified tRNA^fMet^ was acquired at 25°C. The secondary structure of the non-modified tRNA^fMet^ is shown. Unambiguously assigned imino peaks are highlighted in either purple (acceptor stem), blue (D-arm), orange (ACSL) or red (TΨC-arm). (**B**) Chemical shift difference between the imino resonances of the non-modified and modified tRNA^fMet^ construct was calculated as absolute values of the difference between corresponding imino signals (Supplementary Table S1 and Supplementary Table S2). Experimental details are provided in an extended figure caption in SI.

To enable the investigation of tRNA dynamics at nucleotide resolution, we used solution-state NMR spectroscopy to map the base pairing interactions. Therefore, we assigned all imino proton resonances of nucleobases involved in stable hydrogen bonds for both modified and non-modified tRNA^fMet^ (Supplementary Table S1 and Supplementary Table S2). So far, only a subset of signals in the modified tRNA were assigned (82) and to our knowledge there is no non-modified tRNA^fMet^ NMR assignment published yet. Here, we assigned all resonances for the modified tRNA corresponding to 96% of all base-paired residues (Figure 2, Supplementary Figure S3, Supplementary Table S3). The TΨC-arm and the acceptor stem were assigned completely and unambiguously. For the non-modified tRNA three resonances cannot unambiguously be assigned (Figure 3, Supplementary Figure S4).

As tRNA^fMet^ contains the highest content of GC base pairs among all bacterial tRNAs (83), the chemical shift dispersion is reduced leading to resonance overlap in the guanine imino group region. The non-modified tRNA only harbors the four canonical nucleotides and does not contain any modification. This renders the assignment of the non-modified tRNA more challenging than of its modified counterpart. However, the strong NOE cross peak caused by the G64:U50 wobble base pair can be used as a starting point resulting in the complete assignment of the TΨC-arm. Furthermore, the acceptor stem can then be assigned starting from the tertiary interaction G7:G49. By overlaying the spectra of the modified construct, the two guanosine resonances of the D-arm and the three guanosine resonances of the ACSL could be assigned due to an identical pattern. This results in assignment of 72% of base-paired imino groups in the non-modified tRNA (Supplementary Table S3).

Not only the assignment of resonances in the presence of modifications is highly facilitated, but in turn also the mapping of tertiary interactions within the RNA. By NMR spectroscopy it is possible to characterize the interaction network around the modified nucleotides s^4^U8, C_m_32, T54 and Ψ55 and monitor the unstacked residue D20.

In general, comparison of the chemical shifts for the nucleotides in the two tRNA species enables to decipher regions where the introduction of modified nucleotides leads to structural changes. Not unsurprisingly, residues in the proximity of the modifications show the strongest changes in chemical shifts as these residues experience a different chemical environment. However, the most eminent changes are observable for those in the D- and TΨC-arm (Figure 3B).

Thiouridine (s^4^U8), that exhibits prominent chemical shifts of its imino group (δ (^1^H): 14.5 ppm/δ (^15^N): 183 ppm), resides in a base triplet formed with A14 and A21 (s^4^U8:A14:A21). The Watson–Crick Hoogsteen interaction between U8-N3H3 and A14-N7 can be directly mapped via the HNN-COSY experiment through its ^2h^J_N3N7_ coupling (Figure 2C). The interaction between A21 and A14 is only eminent in the respective NOESY through-space connectivity. However, the formation of this interaction can be further corroborated from the close spatial proximity of residue G22 to U8 as eminent from their imino-to-imino cross peak (Figure 2A, Supplementary Figure S3C).

The neighboring residues T54 and Ψ55 are involved in many different non-Watson–Crick like base pairing interactions that are essential to establish the tertiary interaction between the D- and the TΨC-arm. The intra-loop interaction between T54 and A58 is mediated through formation of hydrogen bonds between the T54 Watson-Crick side and the A58 Hoogsteen edge, again detectable with the HNN-COSY experiment (84) (Figure 2C). Further, the NOESY connectivity of T54’s methyl group resonating at 1 ppm (Figure 2A) to G18, G53 and Ψ55 confirms the close proximity of these nucleotides to each other as determined by crystallography and highlights the long-range interaction between nucleotides from the D- and TΨC-arm. Further, the long range interaction established through the Levitt base pair (85) between G15 and C48 can be detected in the spectra of the modified tRNA (Figure 2).

**Figure 4.**
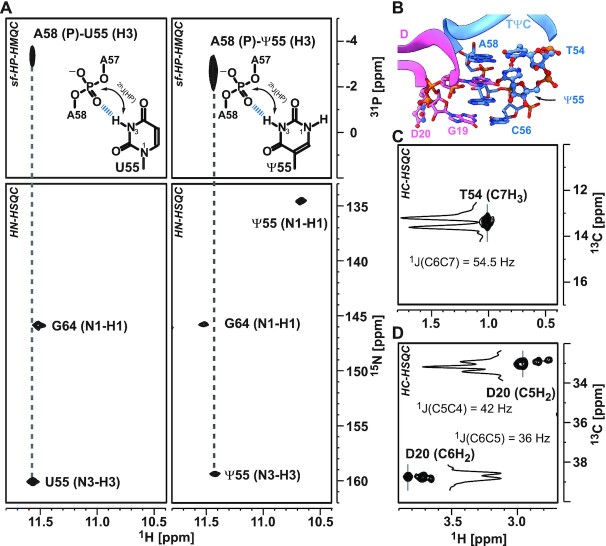
NMR spectroscopic signatures of modified residues. (**A**) Characterization of N3H3-phosphate group hydrogen bonds through sofast-^1^H,^31^P HMQC experiments. The H3 atom of residues U55 (left panel) and Ψ55 (right panel) with the phosphate group of residues A58 is correlated and related to the according HN cross peaks of the corresponding ^1^H^15^N HSQC, which are given in the panels below. (**B**) Placement of modified residues within the core region, which are establishing the interaction between D- (pink) and TΨC-loop (blue) as mapped by crystal structure (PDB: 3CW6). (**C**) The C7H_3_ cross peak of T54 detected in ^1^H,^13^C HSQC and (**D**) the methylene-group cross peaks of D20 C5H_2_ and C6H_2_ in ^1^H,^13^C HSQC. The inserts in (C) and (D) reflect the traces along the ^13^C-dimension to highlight the multiplet structure and reveal the respective ^1^J_CC_ couplings. Experimental details are provided in an extended figure caption in SI.

Within the latter loop a sharp turn in the sugar-phosphate backbone is formed that is stabilized through a hydrogen bond between the N3H3 group of Ψ55 and the OP group of A58. This interaction can be monitored via a long-range HP-correlation experiment (86,87) (Figure 4). Interestingly, the very same interaction between residues U55 and A58 can also be observed in the non-modified tRNA. Furthermore, no rotation of Ψ55’s nucleobase around the C1′–C5 torsion to facilitate a hydrogen bond between N1H1 and the phosphate backbone is observed.

To investigate the base pair stability, we measured proton exchange rates of the imino protons with the surrounding water. Solvent-exchange rates report on the kinetics of the replacement of imino protons with water protons. This exchange process can only occur in open states of base pairs. Thus, higher exchange rates are indicative for less stable base pairing interactions leading to faster exchange kinetics. The reduced solvent-exchange rates of imino groups in dihydrouridines compared to uridines allows the detection of the unstacked and unpaired nucleotide D20 through its HN-correlation (Figure 2B). Besides this, the ethylene group of D20 can easily be assigned in HC-correlation spectra due to the absence of other RNA resonances within this spectral region (Figure 4). Due to its unstacked nature, the line widths of the ethylene group are rather narrow, and the exhibited multiplicity of the peaks are utilized to unambiguously assign the resonances. Interestingly, for each of the CH_2_ groups three distinct peaks with different intensities can be observed. This spectral feature reports on a conformational exchange between at least three distinct structural states for the residue D20.

The 2′-O methyl group of residue C_m_32 is also clearly detectable resonating at δ (^1^H) 3.76 ppm/δ (^13^C) 60.2 ppm in the HC-correlation spectra (Supplementary Figure S3D). It can be used as starting point for the assignment of the ACSL arm. So, the three conserved base pairs G31:C39, G30:C40 and G29:C41 are assigned. Although our assignment is in agreement with the conformational model presented in the crystal structure of the tRNA^fMet^, we do not detect the predicted A38(+)C_m_32 base pair, even at low pH values (pH 5.5) (24).

**Figure 5.**
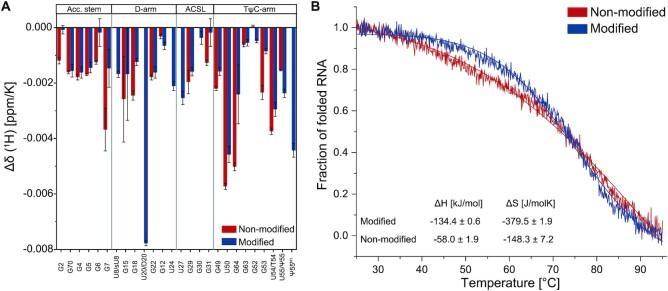
Temperature coefficients of the proton chemical shifts (**A**) and CD melting profiles of the non-modified (blue) and modified (grey) tRNA^fMet^. (A) Temperature coefficients of the proton chemical shifts for the non-modified tRNA ((^1^H), blue bars) and for the modified tRNA^fMet^ construct ((^1^H), red bars). The temperature coefficients were determined through a linear fit of the measured chemical shifts at temperatures ranging from 5 to 45°C (Supplementary Figure S4–S5). (**B**) CD melting profiles were measured and analyzed as described in the method section. The resulting fitting parameters are shown.

With the assigned NMR spectroscopic fingerprints of the modified and non-modified tRNA at hand we can now follow their structural changes with temperatures (Figure 5A, Supplementary Figure S5–S6). Temperature coefficients report on the extend of changes of chemical shifts with changes in temperatures. As the chemical shift is sensitive to the surrounding local structures, larger temperature coefficients can be attributed to larger temperature induced changes in local structure. Regions susceptible to temperature variations are affected to a larger degree and also seem to be more extended in the non-modified than the modified tRNA. This is also reflected in the CD-spectroscopic melting curve where the determined fraction of unfolded RNA is plotted against the temperature (Figure 5B). For the modified tRNA^fMet^, Δ*H* and Δ*S* values of −134.4 ± 0.6 kJ/mol and −379.5 ± 1.9 J/mol K were determined, respectively; in comparison, −58.0 ± 1.9 kJ/mol and −148.3 ± 7.2 J/mol K were determined for the non-modified tRNA^fMet^. Here, a significant decrease in helicity — as a measure for the foldedness of the tRNA — is observed already at ambient temperatures (>30°C). This behavior indicates an earlier melting of tertiary interactions in the non-modified compared to the modified tRNA, whereas — under the chosen conditions (200 mM K^+^ and 5 mM Mg^2+^, pH 6.2) — the individual secondary structures are of comparable stability.

### Local stability of tRNA

In order to analyze the local dynamics of tRNA^fMet^, we measured ^15^N spin relaxation data and solvent-exchange rates (Supplementary Tables S4–S7, S13–S14 and Supplementary Figures S7–S10).

Spin relaxation data and solvent-exchange rates are sensitive to dynamics on different motional timescales (74,88,89). Spin relaxation data of nuclei such as ^13^C and ^15^N contain information about the dynamics in the range of ps to ns through modulation of the underlying spin interactions (88). On the other hand, the solvent exchange rates represent the exchange between solvent protons and imino protons based on base pair opening and base flipping on a ms timescale (74,89).

The relaxation data were collected for the modified and non-modified tRNA at two magnetic fields (600 MHz and 800 MHz) and for one field at two different temperatures (25°C and 40°C) (Supplementary Figures S7–S8). The relaxation data are characterized by the longitudinal *R*_1_ rate, the transversal *R*_2_ rate and the hetNOE as an indication for cross-relaxation between ^1^H and ^15^N. Unsurprisingly, the *R_1_* values for both tRNAs increase with higher magnetic field from 0.4 rad/s to 0.6 rad/s, where the non-modified tRNA exhibit a larger increase than the modified tRNA. The transversal *R*_2_ rate is determined to around 20 rad/s, which is constant at both magnetic fields for the modified tRNA, but increases up to 30 rad/s for the non-modified construct. This trend is also reflected in the *R*_2_*/R*_1_ ratio, which is constant between 30 and 60 for the modified tRNA at both magnetic fields, but increases from 30 at 600 MHz up to 90 at 800 MHz in the case of the non-modified tRNA. In contrast, the hetNOE shows values between 0.6 and 0.9, which is expected for a sizeable RNA. Furthermore, it shows no large deviation between both magnetic fields and tRNAs. At an elevated temperature of 40°C, the temperature-induced decrease of the hetNOE and *R*_2_*/R*_1_ ratio is observed showing the temperature-induced flexibility of the tRNAs. However, no larger deviation was observed between both tRNAs.

In order to compare the three different datasets in more detail we performed a model-free analysis (65,66) with the program RELAX (Supplementary Table S8-S12) (67,68). As expected for a structured RNA, the ${S^2}$ order parameters range between 0.75 and 0.98 for both tRNAs. In general, the ${S^2}$ order parameter is a normalized indication for the flexibility, where ${S^2} = \ 0$ corresponds to a highly flexible and a ${S^2} = \ 1$ is equal to a highly rigid bond vector. For both tRNAs similar global rotational correlation times which indirect reflects the molecular shape of either 17.4 ns (non-modified) or 20.4 ns (modified) and an isotropic diffusion value of the axially symmetric tensor on the order of 10^7^ s^–1^ were determined. The determined values are expected for a large RNA molecule with a molecular mass of 26 kDa (89–91) and reflect an overall identical shape of both molecules (Table 1).

**Table 1.** Description of the diffusion tensor obtained by the model-free analysis of the ^15^N relaxation parameters. ^15^N spin relaxation data of non-modified and modified tRNA^fMet^ were analyzed with the program RELAX (67,68) resulting in the diffusion tensor and the *model-free* parameters (Supplementary Table S8-S12) of both RNAs. The parameters and the explanation of the diffusion tensor are given in the first two columns, the determined value at the given temperature in the following columns

		Unmodified tRNA^fMet^		Modified tRNA^fMet^	
		25°C	40°C	25°C	40°C
Rotational correlation time	τ_m_ [ns]	17.4	9.05	20.4	13.0
Isotropic part of the diffusion tensor	*D*_iso_ [10^6^ rad/s]	9.56	18.4	8.19	12.8
Anisotropic part of the diffusion tensor	*D*_a_ [10^6^ rad/s]	5.94	11.2	−6.04	−8.22
Ratio between parallel to orthogonal part of the diffusion tensor	*D*_ratio_	1.78	1.76	0.41	0.47
Spherical angles describing the orientation of the main axis in the diffusion tensor	Theta [rad]	0.59	0.59	1.90	1.90
	Phi [rad]	1.57	1.57	2.95	2.95

Out of the ten models (Supplementary Table S8), the best models of the model-free analysis describing the ^15^N relaxation parameter are listed in Supplementary Table S9-S12. The models are characterized with an increasing complexity starting by the simplest model M0, and ends in the most complex model M8 which is described by five parameters. Hereby, models M0, M1 and M9 assume very fast local dynamics (τ_f_ < 10 ps) with either no further parameter, the *S*^2^ order parameter or an additional *R_ex_* contribution on the ms timescale. Further complexity of the models is introduced with a local correlation time τ_e_ of the group of interest representing the timescale of the motion. The models M5 to M8 describe a more complex behavior of the imino group. Here, the order parameter as well as the local correlation time are divided into a fast (τ_f_ < 100 ps) and a slow (τ_s_ > 100 ps) motion assuming that the group of interest is involved both in a very fast and a slower dynamic motion.

Thereby, the ^15^N spins of the imino group in the non-modified tRNA show a strong tendency to motion models which involve the exchange parameter *R*_ex_ at 25°C. At 40°C, the selected models of the non-modified tRNA comprises only simpler ones such as M1 or M2 suggesting a potential melting of the exchange states. On the contrary, models comprising no exchange were selected for almost all ^15^N spins of the modified tRNA (models M2, M4–7) suggesting one stable conformational state with slow global dynamics and a few motional hotspots.

**Figure 6.**
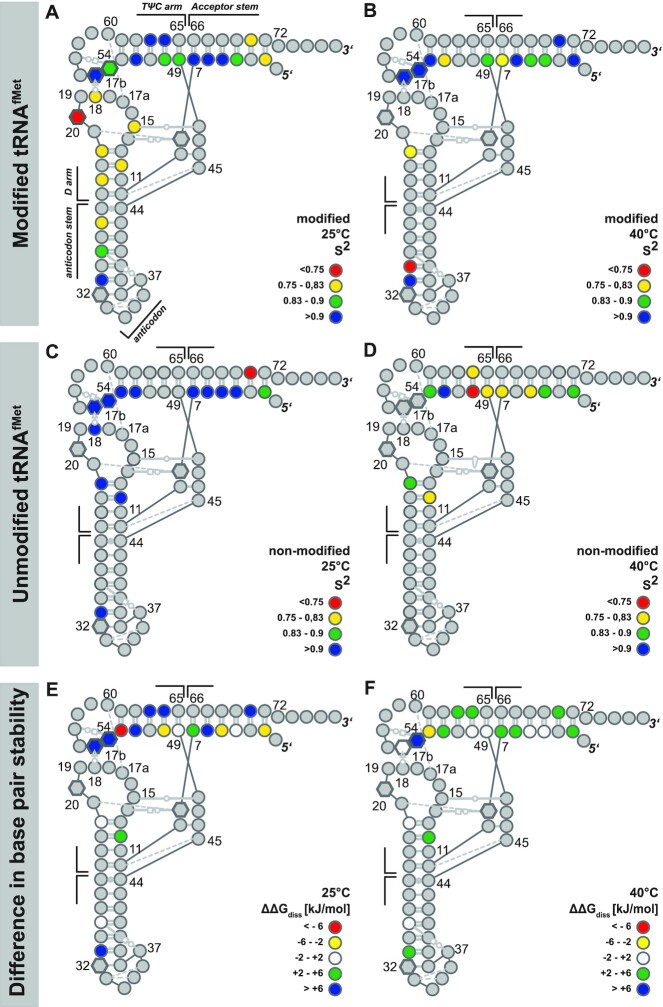
Comparison of local dynamics and base pair stabilities for modified and unmodified tRNA^fMet^. Nucleotide-resolved representation of the L-shaped structure that is color coded to represent the S^2^ order parameter for (**A**) native tRNA at 25°C and (**B**) at 40°C; (**C**) unmodified tRNA at 25°C and (**D**) at 40°C. The base interactions are annotated according to the nomenclature of Leontis and Westhof (112), modified residues are represented by hexagons instead of circles. The differences in the stabilities of corresponding base pairs ($\Delta \Delta {G_{diss}} = \Delta {G_{diss}}^{modified}\ -\ \Delta {G_{diss}}^{non - modified}$) are given for (**E**) 25°C and (**F**) 40°C. Positive values shown in blue and green mean that modified tRNA is more stable than unmodified tRNA. In contrast, negative ${\rm{\Delta \Delta }}{G_{diss}}$ values shown in yellow and red show that unmodified tRNA is more stable than modified tRNA. White highlighted nucleotides represent only a minor difference in ${\rm{\Delta \Delta }}{G_{diss}}$ between both tRNAs. All data is summarized in Supplementary Tables S9-S12 and S15-S16.

For the native, modified tRNA we find that at 25°C, judging from the order parameters, the local dynamics in the anticodon domain (anticodon-stem and D-arm) are generally higher compared to those in the TΨC and acceptor stem (Figure 6A). This indicates that the local dynamic features are similar for the pairs of coaxial stacked helices in the tRNA^fMet^.

In the acceptor stem only residues close to the CCA-end exhibit increased local dynamics. Residue D20 is the most dynamic on the fast time-scale, presumably because it is not involved in stacking interactions with any other residue. Increasing the temperature to 40°C results in a general increase in local dynamics of the modified tRNA (Figure 6B). This is especially manifested for residues residing in the helical stacking region of TΨC and acceptor stem and further for those in the anticodon stem close to the anti-codon loop. However, at the elbow region of the native tRNA where the D- and TΨC-arm interact, the local dynamics are not increased (T54, Ψ55).

The non-modified tRNA exhibits at 25°C rather uniform low dynamics with *S*^2^ > 0.9 (Figure 6C). At this temperature, only residues close to the CCA-end in the acceptor stem exhibit increased local fluctuation on the fast timescale, as has been already observed for the modified tRNA. This behavior changes at elevated temperatures (Figure 6D). For nearly all residues in the non-modified tRNA, a decrease in the order parameter and hence an increase in local dynamics is observable at 40°C. Similar to the modified tRNA, the largest increase is manifested at the helix junction. However, for the elbow region a different behavior is observed, in contrast to the native tRNA, the transcribed tRNA does not exhibit a relative decrease of dynamics in the elbow region upon increased temperature.

Comparing the dynamics of tertiary interactions represented by the nucleotides D20, G18, T54 and Ψ55 to secondary interactions (e.g. the acceptor stem), only minor differences between these interactions can be found in the *S*^2^ order parameter and the local correlation time *τ*_e_ for both tRNAs at 25°C. At a temperature of 40°C, a difference between secondary and tertiary interactions is observable. In case of the modified tRNA, this difference is represented by the timescale of the local dynamics, which is on the ns timescale for the secondary interactions and on the ps timescale for tertiary interactions. In contrast, the tertiary interactions were not detectable for the non-modified tRNA due to melting of the tertiary structure.

The comparison of the exchange rate *R*_ex_ being a measure for conformational exchange on the slow ms timescale indicates a more stable conformation for the modified tRNA^fMet^ (Supplementary Table S9-S12). We report that at a temperature of 25°C only five imino groups in the modified tRNA exhibit a conformational exchange according to the model-free results with largest contributions in the D-arm possibly due to the impact of D20, whereas 12 imino groups evenly distributed over the sequence feature conformational exchange in the non-modified tRNA. However, increasing the temperature to 40°C results in a completely different situation. In the modified tRNA, conformational exchange is now found for imino groups in the TΨC-arm rather than in the D-arm, albeit with similar *R*_ex_ rates. Interestingly, the non-modified tRNA exhibits no *R*_ex_ rates at all at this temperature. This suggests that the conformational exchange being observable at room temperature for the non-modified tRNA ceases and only one conformation is found at elevated temperatures. In the case of the modified tRNA, the shift of conformational exchange on the ms timescale from D-arm to TΨC-arm could be explained with the beginning of melting of tertiary interactions and thus a destabilized conformation at the elbow region. Notably, this trend follows the CD-spectroscopic melting curves showing an initial decrease of folded RNA fraction for the non-modified tRNA (Figure 5B, blue line).

Similar to the relaxation data, the solvent-exchange rates of imino protons report on the stability and hence local base-pairing dynamics (74). From the temperature profile of solvent exchange rates, the thermodynamic parameters of base pair opening can be deduced. The observed base-pair stabilities range at 25°C between 20.9 and 47.4 kJ/mol for the native tRNA and between 15.0 and 32 kJ/mol for the non-modified tRNA. In the range between 20 and 40°C, the native tRNA exhibits very stable base pairs and those are mostly more stable than the corresponding base pairs within the non-modified tRNA; this is indicated by the difference of the free dissociation enthalpy ${\rm{\Delta \Delta }}{G_{diss}}$ between the modified and non-modified tRNA (Figure 6E and F). Thereby, a positive value of ${\rm{\Delta \Delta }}{G_{diss}}$ corresponds to a more stable base pairing in the modified tRNA. It is interesting to note that the modified tRNA has a high enthalpic contribution to base-pair stability whereas the non-modified tRNA exhibits comparably low base-pair dissociation enthalpy but mostly positive dissociation entropy (Figure 5B, Figure 6E and F, Supplementary Tables S15 and S16). Notably, temperature increases are less impactful for the native than for the unmodified tRNA. At elevated temperatures, the gross of base pairs is more stable in the modified compared to the unmodified tRNA; especially the base- pairing stabilities in the elbow region and at the helix junctions exhibit a higher relative stability.

## DISCUSSION

The modifications in tRNAs are numerous and modulate the recognition and binding of their cellular interaction partners like aminoacyl-tRNA synthetases (aaRS), mRNAs and ribosomes (92). Although modifications in tRNAs are highly abundant, they are not equally distributed over their conserved structure but occur clustered within the structural core and the anticodon stem loop (ACSL) (93). Within the full-length initiator tRNA^fMet^ from *E. coli* produced by overexpression in minimal medium the core modifications (s^4^U8, D20, T54, Ψ55) are incorporated almost to completion (85–101% ± 1%) whereas the modification in the ACSL is only found in a subset of the molecules. In the presence of 5 mM Mg^2+^-ions the modified tRNA exhibits a higher structural stability as its non-modified counterpart, whose tertiary interactions melt earlier as evidenced by CD-melting experiments. This is also represented in the larger (more negative) Gibbs free energy for the modified tRNA in the ambient temperature range. Based on crystallographic structures and solution state NMR experiments, it is expected that modified tRNA and non-modified tRNA have a similar global structure (36,37,40,43,94,95). Through assignment of the imino groups of both forms of tRNA^fMet^ as well as the carbon resonances of the introduced modifications we could confirm the predicted secondary and tertiary interactions, with exception of the protonated base pair in the anticodon stem-loop. This could be an indication that in the crystal structure of the tRNA^fMet^ a conformation of the ACSL is frozen out which is under ambient conditions only populated to a small extent (<10%), rendering it invisible in standard NMR experiments employed here (96).

Due to the different chemical environments, the chemical shifts of the imino groups vary in regions where nucleobases are modified. Moreover, very large differences can be found for all core modifications whereas the ACSL modification C_m_32 has a smaller influence on the chemical shifts of the nearby imino groups. From earlier theoretical and experimental works it is deduced that modifications restrict the conformational flexibility of the tRNA (10,97–100). However, the dynamic behavior of nucleotides in tRNA^fMet^ is quite different. At 25°C the non-modified tRNA seems to be less dynamic than the modified counterpart as the former exhibits on average slightly higher order parameters. This more rigid-like behavior on the fast ps- to ns-timescale is complemented by exchange reactions between conformational substates found in 12 residues of the non-modified tRNA. This conformational exchange, however, has to occur on the slower μs- to ms-timescale. Furthermore, similarly in the modified tRNA, five residues show conformational exchange, and for the resonance of D20 three distinct peaks are observed in the [^1^H,^13^C]-HSQC spectrum, also indicating exchange between three different conformational substates. Upon temperature increase to 40°C, there are no significant changes in the order parameters and thus also no changes in the local dynamics of the nucleotides in the modified tRNA. In contrast, for the non-modified tRNA, the order parameters drop significantly upon temperature increase and thus the dynamics tremendously increase. This is supported by the CD-spectroscopic melting profiles, which show for the modified tRNA almost no decrease until a temperature of about 50°C; thus it has a more temperature-stable tertiary structure. Both findings agree with the dynamics occurring on a slower time scale as investigated via the solvent-exchange rates. Here, the base pairs of the modified tRNA^fMet^ are typically around 6 kJ/mol more stable than in the non-modified tRNA at temperatures between 25°C and 40°C. Above 50°C, no difference in (base-pair) stability due to simultaneous melting of secondary structure elements can be determined. Similarly, for tRNA^Val^ it was found by HD-exchange rates (34) that the local base pair stabilities of selected nucleotides are higher. In summary, these findings lead to two main conclusions: (i) Although the modifications for the tRNA^fMet^ are clustered in the core region, a remote effect of stabilization throughout all base-pairs of the whole molecule is observed. This is in line with earlier findings that showed that modifications affect correlated motions within tRNAs (101), a mechanism that could mediate the overall stabilizing effect of nucleotide modifications throughout the molecule. (ii) From the differential temperature profile of order parameters and the higher degree of fast dynamics at 25°C, the stabilization of the modified tRNA has to be attributed to entropic effects. In other words, increased fast local conformational dynamics lead to a stabilization of the tRNA’s tertiary structure. Such local structural fluctuations were also observed in molecular dynamic simulations (34,103), however they are not manifested as differences in the tertiary structures of the various tRNA^fMet^ solved so far (Supplementary Figure S11). Importantly, the local dynamics do not negatively impact the tertiary structure of the tRNA as the global structure remains intact. The introduction of modifications can therefore regarded to be essential for a harmonization of the stabilities of secondary and tertiary structure elements in tRNA^fMet^.

In this respect, an interesting example is the modification of uridine to dihydrouridine in the D-loop that is highly conserved in all three domains of life. Consistent with reported results (19), our analysis finds D20 as the most flexible nucleotide in the modified tRNA. At 25°C it exhibits a low ${S^2}$ order parameter of 0.52 and additionally can be observed to exist in at least three different conformational substates which may only exchange on the slower timescale. Such low order parameters are typically found for nucleotides which are highly flexible, such as looped nucleotides pointing towards the solvent surface, e.g. the second nucleotide in YNMG loops (91). Interestingly, the D20 nucleotide is located close to those modified nucleotides which establish the tertiary interactions responsible for the formation and stabilization of the elbow region of the L-shaped structure (T54 and Ψ55) (104). Our analysis shows that pseudouridylation introduces stability to the tertiary structure as we cannot detect a signal for U55 in the non-modified tRNA at higher temperatures (>45°C). In line with reported results for tRNA folds, the rigidity of Ψ55 and nearby nucleotides is maintained with an ${S^2}$ order parameter of 0.90 and 0.93 at 25 and 40°C, respectively. To date, the impact of Ψ is highly discussed (33). In general, it is shown that Ψ stabilizes tRNA folds but in other RNA the stabilizing effect is not shown with clear evidence. For the tRNA^fMet^ here, we assume that the interplay of flexibility of D20 and the stabilization of Ψ55 is needed to stabilize the very important elbow region of the L-shape, while Ψ acts through enthalpic and D through an entropic mechanism. Besides the increased thermodynamic stability that might be of biological importance during encounters of environmental stresses, the enhanced local dynamics as evidenced for D20 and other residues will play a role during functional interactions. For example, during translation initiation the tRNA^fMet^ must retain its overall shape while allowing a certain degree of flexibility during correct accommodation at the right position within the 30S subunit and towards the start codon at the mRNA (21,105,106). A delicate interplay of rigidity and flexibility is necessary. While at the same time rigidity of the anticodon stem is required for translocation during translation, structural fluctuations are required for building the correct codon:anticodon (102). Functional evidence that the nucleotide modifications are causal for the balance of local fluctuations and overall stability is provided by ribosome binding studies where the ${k_{on}}$ values are identical, but the ${k_{off}}$ values for unmodified tRNAs are largely increased compared to those containing modifications (107,108). Besides ribosome binding, the modifications’ impact on local flexibility can also play a crucial role in the accuracy and fidelity of translation (108,109).

So far, it was shown that modified residues can either increase the rigidity and thermal stability of the structure of tRNA or have exactly the opposite effect (10,110). Until a similar comparative mapping of local structural dynamics is undertaken for more full length tRNAs, it remains to be seen if the findings provided here for tRNA^fMet^ are generally applicable to all tRNAs. It will be especially insightful to understand how the local dynamics interfere, modulate and direct the incorporation of the modified nucleotides themselves (111).

## DATA AVAILABILITY

Chemical shifts and relaxation data are deposited at BMRB under accession code 51144.

## Supplementary Material

gkac040_Supplemental_File
