# Supplementary material for: RNA modifications stabilize the tertiary structure of tRNAfMet by locally increasing conformational dynamics
Source: Nucleic Acids Res. 2022 Feb 7;50(4):2334–49. doi: 10.1093/nar/gkac040 (PMC8887418; doi:10.1093/nar/gkac040)
Supplement: gkac040_Supplemental_File [file gkac040_supplemental_file.pdf]

Supplementary Material

RNA modifications stabilize the tertiary structure of $tRNA^{fMet}$ by locally increasing conformational dynamics

Thomas Biedenbänder[1,2†], Vanessa de Jesus[1†], Martina Schmidt-Dengler[3], Mark Helm[3], Björn Corzilius[2] and Boris Fürtig[1,*]

1 Institute for Organic Chemistry and Chemical Biology, Center for Biomolecular Magnetic Resonance (BMRZ), Johann Wolfgang Goethe-Universität, Frankfurt am Main, 60438, Germany

2 Institute of Chemistry and Department Life, Light & Matter, University of Rostock, Rostock, 18059, Germany

3 Institut für pharmazeutische und biomedizinische Wissenschaften (IPBW), Johannes Gutenberg-Universität Mainz, 55128, Germany

* To whom correspondence should be addressed. Tel: +49 69 7982 9157; Fax: +49 69 7982 9515; Email: fuertig@nmr.uni-frankfurt.de,

† These authors contributed equally to this work.

Extended Figure Caption:

Figure 2: Selected [^{1}H,^{1}H]-strips of a 3D-[^{1}H,^{15}N,^{1}H]-SOFAST-HMQC-NOESY (A), 2D-[^{1}H,^{15}N]-BEST-TROSY (B) and 2D-[^{1}H,^{15}N]-BEST-TROSY-HNN-COSY experiment (C) of modified tRNA^{fMet} at 25 °C. (A) NOESY spectra were acquired at a field strength of 900 MHz. Each [^{1}H,^{1}H] strip has the chemical shift of the indicated imino resonance. All imino resonance are listed in Supplementary Table S1. The spectrum was recorded with 96 scans and 60 points and 128 point in the second and third indirect dimension, respectively. Carrier frequencies were set to 4.698 ppm (^{1}H) and 153.25 ppm (^{15}N). The spectral width was set to 25.244 ppm (^{1}H dimension), 19.5 ppm (second ^{15}N dimension) and 15.76 ppm (third ^{1}H dimension). Radiofrequency of the hard pulses were set to 15.03 kHz (^{1}H) and 6.49 kHz (^{15}N). The relaxation delay was set to 0.3 s and the acquisition time was set to 45.1 ms. Garp4.p62 was used as a decoupling scheme during acquisition. (B) The 2D-[^{1}H,^{15}N]-BEST-TROSY experiment of the modified construct was recorded with 16 scans and 400 points in the indirect ^{15}N dimension. Carrier frequencies were set to 4.698 ppm (direct dimension) and 159 ppm (^{15}N). The spectral width was set to 25.24 ppm (direct dimension), and 55 ppm (indirect dimension). Radiofrequency of the hard pulses were set to 15.03 kHz (^{1}H). The hard pulse for 15N decoupling was set to 6.49 kHz. The relaxation delay was set to 0.1 s and the acquisition time was set to 45.1 ms. Garp4 was used as a decoupling scheme during acquisition. (C) Spectrum of 2D-[1H,15N]-HNN-COSY experiment of the modified tRNA^{fMet} construct (210 µM). The spectrum was recorded with 256 scans and 768 points in the indirect dimension. Carrier frequencies were set to 4.697 ppm (1H) and 190 ppm for the indirect dimension (15N). The spectral width was set to 25 ppm (1H dimension), and 100 ppm (15N dimension). Radiofrequency of the hard pulses were set to 25.77 kHz (1H) and 6.25 kHz (15N). The relaxation delay was set to 0.3 s and the acquisition time was set to 80 ms. Garp4 was used as decoupling scheme during acquisition. Selected regions of the HNN-COSY were multiplied by ten times to highlight less intense peaks. Secondary structure of the modified tRNA^{fMet} is shown in (C). Unambiguously assigned imino peaks are highlighted in either green (acceptor stem), blue (D-arm), brown (ACSL), or red (TΨC-arm).

Figure 3: 2D-[^{1}H,^{15}N]-BEST-TROSY experiment of unmodified tRNA^{fMet} with secondary structure (A) and analysis of chemical shift differences between modified and non-modified tRNA (B). (A) The BEST-TROSY spectrum of the non-modified tRNA^{fMet} was acquired at a magnetic field strength of 700 MHz at 25 °C. The experiment was recorded with 64 scans and 256 points in the indirect ^{15}N dimension. Carrier frequencies were set to 4.698 ppm (direct dimension) and 153.8 ppm (^{15}N). The spectral width was set to 25 ppm (direct dimension), and 22.8 ppm (indirect dimension). Radiofrequency of the hard pulses were set to 14.59 kHz (^{1}H). The hard pulse for ^{15}N decoupling was set to 7.14 kHz. The relaxation delay was set to 0.3 s and the acquisition time was set to 58.6 ms. Garp4 was used as a decoupling scheme during acquisition. Secondary structure of the non-modified tRNA^{fMet}. Unambiguously assigned imino peaks are highlighted in either purple (acceptor stem), blue (D-arm), orange (ACSL), or red (TΨC-arm). All imino resonance are listed in Supplementary Table S2. (B) Chemical shift difference between the imino resonances of the non-modified and modified tRNA^{fMet} construct was calculated as absolute values of the difference between corresponding imino signals (Supplementary Table S1 and Supplementary Table S2).

Figure 4: 2D-[^{1}H,^{31}P]-sofast HMQC spectra were acquired at a magnetic field strength of 700 MHz equipped with a QCI cryo-probe at 25 °C. They were recorded with 16k scans and 32 points in the indirect ^{31}P dimension. Carrier frequencies were set to 4.698 ppm (^{1}H), 154ppm (^{15}N) and 0 ppm (^{31}P). The spectral width was set to 25 ppm (direct dimension), and 20 ppm (indirect dimension). The magnetization transfer was set to $1/(2*J_{HP})$ = 16.6 ms. The relaxation delay was set to 0.5 s and the acquisition time was set to 58.4 ms. Garp4 (^{15}N) and Garp (^{31}P) was used as a decoupling scheme during acquisition.

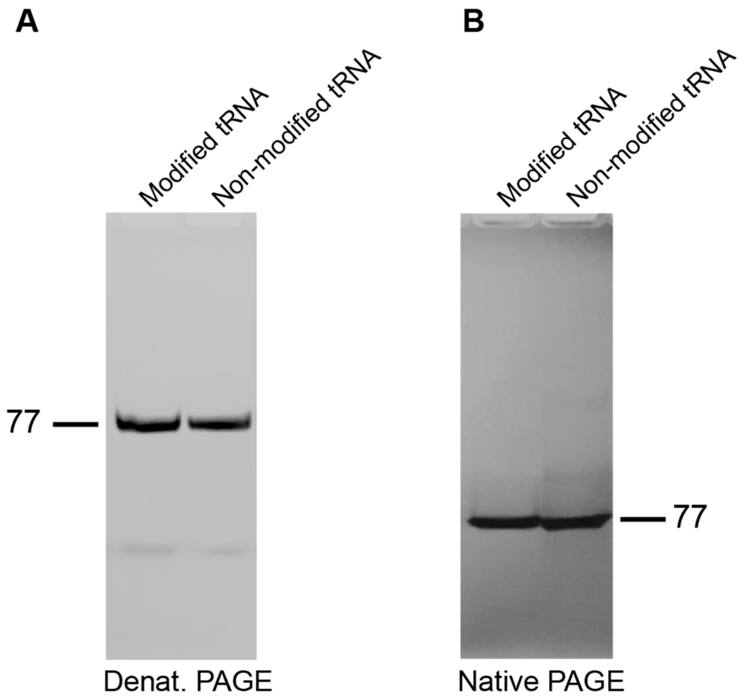

Supplementary Figure S1: Analysis of purified tRNA^{fMet} by 15% denaturing PAGE and 10% native PAGE. For the denaturing PAGE, 160 pmol of modified and non-modified tRNA, respectively, were applied and separated at 180 V at 25 °C for 1 h. For the native PAGE, 300 pmol for each RNA construct was applied and separation was performed at 0.8 W at 25 °C for 1.5 h. RNA molecules in both gels were visualized through UV shadowing at 253 nm.

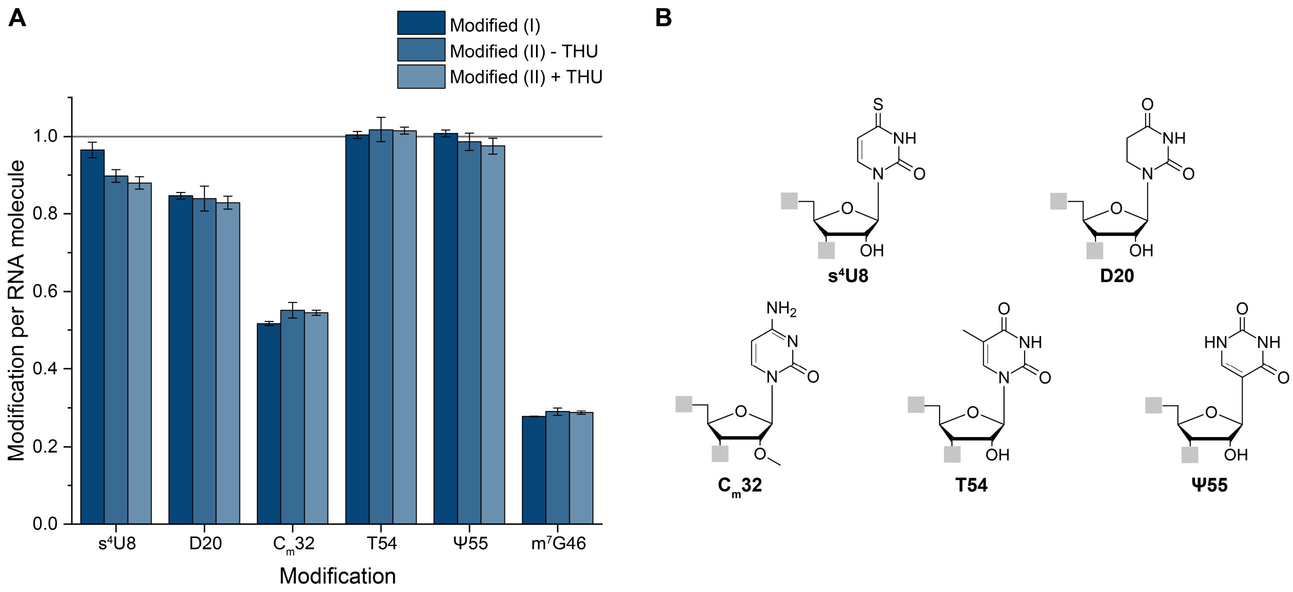

Supplementary Figure S2: LC-MS based quantification of modified nucleosides levels in $tRNA^{fMet}$. (A) All modified $tRNA^{fMet}$ samples were digested to nucleosides and analyzed via LC-MS. Hereby, modified (I) $tRNA^{fMet}$ is the same sample as shown in Fig. 1. The experiments were repeated with respect to the sample preparation for analysis. The samples were either digested in absence (Modified (II) – THU) or in presence (Modified (II) + THU) of tetrahydrouridine (THU) to test the impact of THU on C_m32 analysis. Modified (II) – THU represents the result of the same digestion protocol as Modified (I) tRNA. The amount of each modification was normalized to the amount of injected RNA molecules. The occurrence of one modified nucleoside per RNA molecule is highlighted with a black line. The modified nucleosides are s^4U8 (thiouridine 8), D20 (5,6-dihydrouridine 20), C_m32 (2'O-methylcytidine 32), T54 (ribothymidine 54), Ψ55 (pseudouridine 55), and m^7G46 (7-methylguanosine 46). The measured relative abundance for m^7G46 is based on the endogenous isoacceptor of $tRNA^{fMet}$ in *E. coli.* In *E. coli* K strains the initiator $tRNA^{fMet}$ is encoded by four genes that differ by a single nucleotide at position 46 resulting in two $tRNA^{fMet}$ species[1,2]: metZ, metW and metV encode $tRNA^{fMet1}$ with m^7G46 and metY encode $tRNA^{fMet2}$ with A46. Therefore, the cellular $tRNA^{fMet}$ pool consists of 75% $tRNA_{G46}^{fMet1}$, and 25% of $tRNA_{A46}^{fMet2}$ due to the presence of three genes encoding for m^7G46 and only one gene encoding for A46[2,3]. In contrast, the results here show only relative abundance of 27.7% and thus, the second isoacceptor reflecting the $tRNA_{A46}^{fMet2}$ of interest is successfully overexpressed. (B) Chemical structures of analyzed nucleosides.

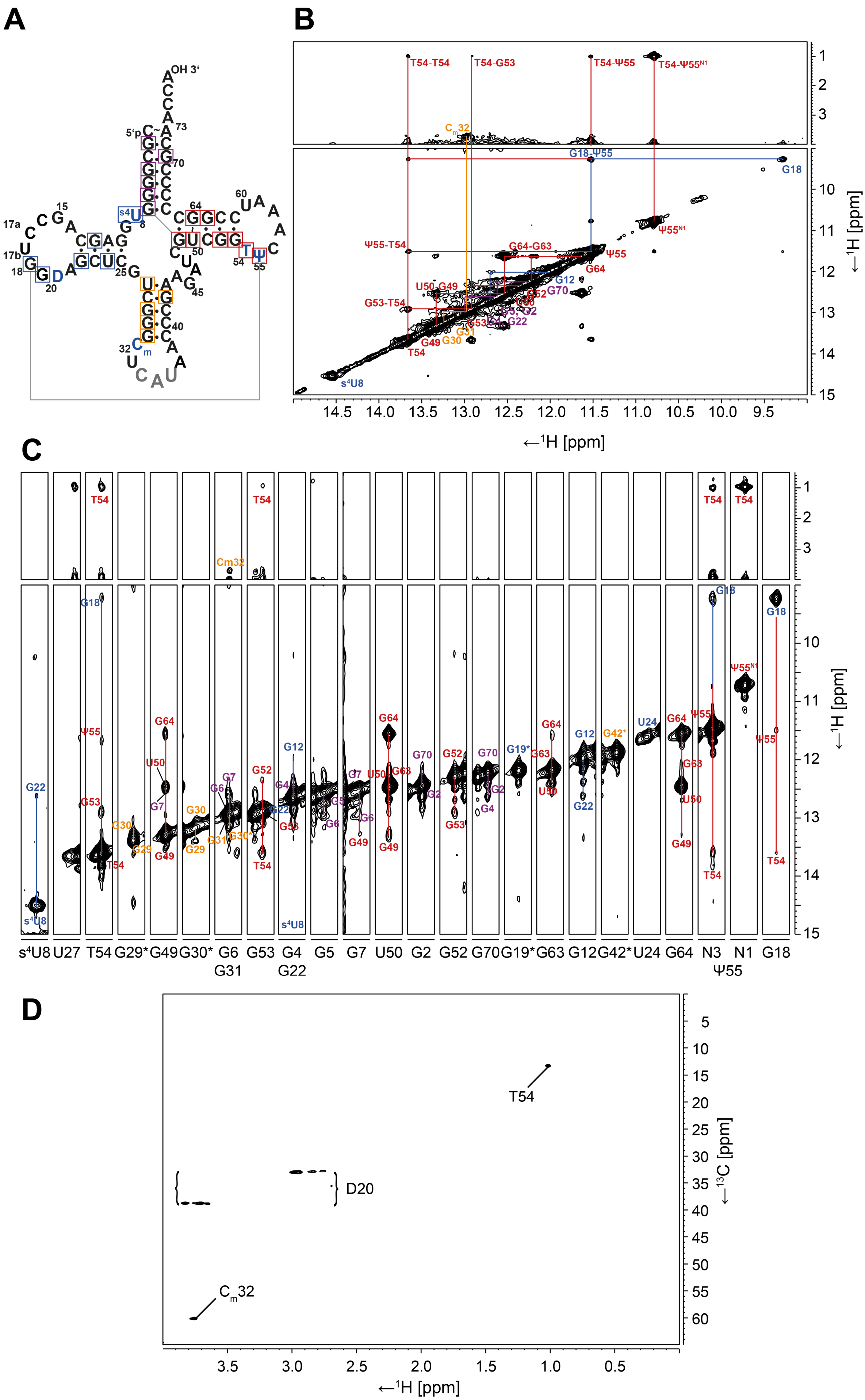
A
B
C
D
T54-T54
T54-G53
T54-Ψ55
T54-Ψ55N1
G18-Ψ55
G18
Ψ55N1
Ψ55-T54
G64-G63
Ψ55
G64
U50-G49
G53-T54
s4U8
←1H [ppm]
←13C [ppm]
s4U8 U27 T54 G29* G49 G30* G6 G31 G53 G4 G22 G5 G7 U50 G2 G52 G70 G19* G63 G12 G42* U24 G64 N3 Ψ55 N1 G18
T54
D20
Cm32

Supplementary Figure S3: NOESY spectra (B, C) and [^{1}H,^{13}C]-HSQC spectrum (D) of the modified tRNA^{fMet} construct (531 µM) acquired at a field strength of 600 MHz at 290 K (B) and 900 MHz at 298 K (C,D). The buffer contained 25 mM KPi (pH 6.2), 200 mM KCl and 5 mM $MgCl_2$. (A) Secondary structure of the modified tRNA^{fMet} from *E. coli*. Unambiguously assigned imino peaks are highlighted in either green (acceptor stem), blue (D-arm), brown (ASL), or red (TΨC-arm). (B) 2D-[^{1}H,^{1}H]-NOESY experiment acquired at a field strength of 600 MHz at 290 K. The spectrum was recorded with 144 scans and 128 points in the second. Carrier frequencies were set to 4.698 ppm (direct dimension) and 160 ppm (^{15}N). The spectral width was set to 25 ppm (^{1}H dimension), and 16 ppm (indirect dimension). Radiofrequency of the hard pulses were set to 18.38 kHz (^{1}H). The hard pulse for ^{15}N decoupling was set to 6.25 kHz. The relaxation delay was set to 2 s and the acquisition time was set to 68.3 ms. Garp4 was used as a decoupling scheme during acquisition. (C) 3D-[^{1}H,^{15}N,^{1}H]-SOFAST-HMQC-NOESY experiment acquired at a field strength of 900 MHz at 298 K. Each strip has the corresponding chemical shift of the indicated imino resonance. All imino resonances are listed in Supplementary Table S1. The spectrum was recorded with 96 scans and 60 points and 128 points in the second and third indirect dimension, respectively. Carrier frequencies were set to 4.698 ppm (^{1}H) and 153.25 ppm (^{15}N). The spectral width was set to 25.244 ppm (^{1}H dimension), 19.5 ppm (second ^{15}N dimension) and 15.76 ppm (third ^{1}H dimension). Radiofrequency of the hard pulses were set to 15.03 kHz (^{1}H) and 6.49 kHz (15N). The relaxation delay was set to 0.3 s and the acquisition time was set to 45.1 ms. Garp4.p62 was used as a decoupling scheme during acquisition. (D) The spectrum for the modified construct was recorded with 8 scans and 256 points in the indirect dimension. Carrier frequencies were set to 4.7 ppm (^{1}H) and 32.5 ppm. The spectral width was set to 16.33 ppm (1H dimension), and 65 ppm. Radiofrequency of the hard pulses were set to 15.03 kHz (^{1}H, modified), and 21.37 kHz (^{13}C). The relaxation delay was set to 1 s and the acquisition time was set to 69.6 ms. For all experiments, Garp4 was used as a decoupling scheme during acquisition.

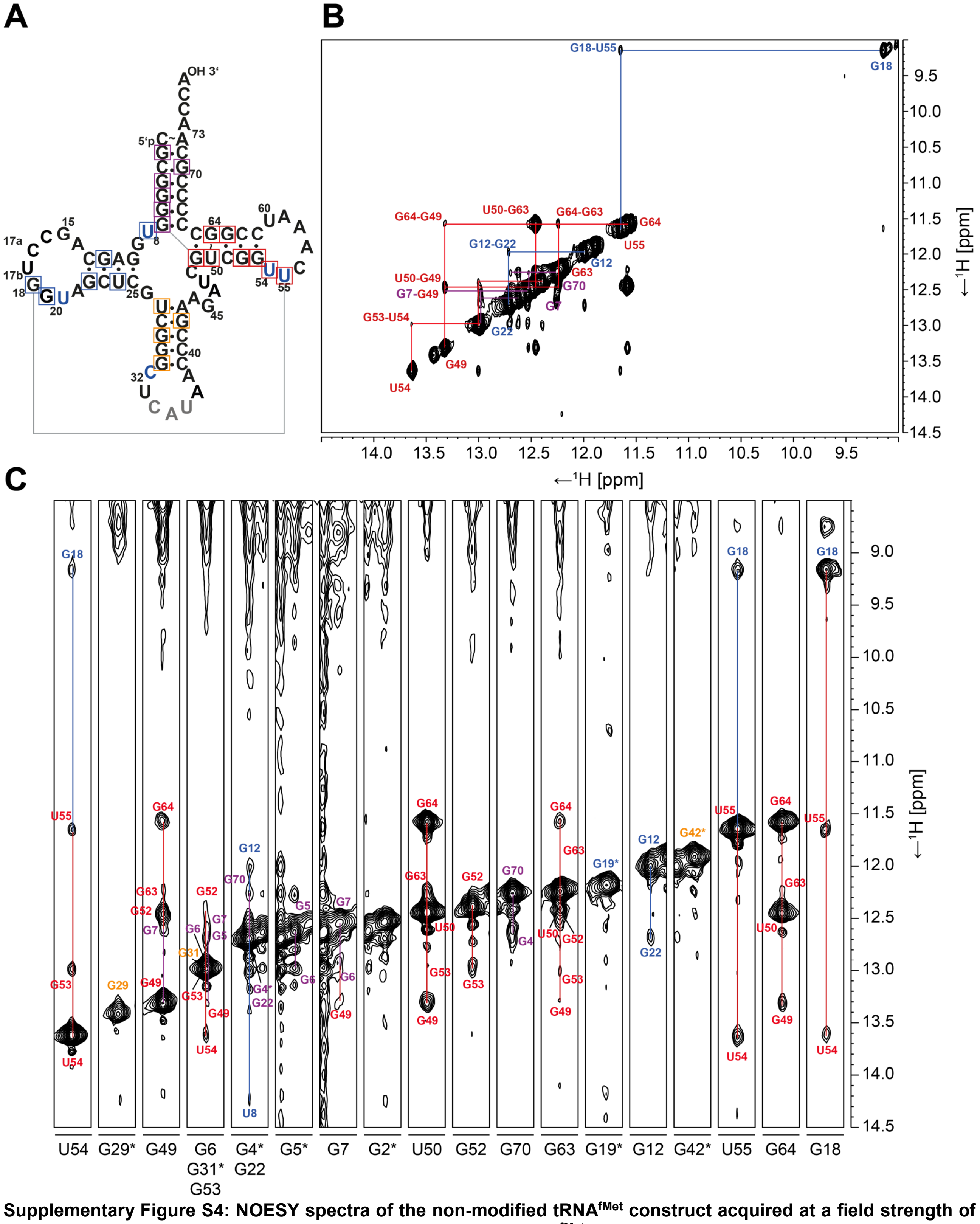

Supplementary Figure S4: NOESY spectra of the non-modified tRNA^{fMet} construct acquired at a field strength of 950 MHz at 298 K. (A) Secondary structure of the modified tRNA^{fMet} from *E. coli*. Unambiguously assigned imino peaks are highlighted in either green (acceptor stem), blue (D-arm), brown (ASL), or red (TΨC-arm). (B) 2D-[^{1}H,^{1}H]-NOESY experiment acquired with a sample concentration of 500 µM from previous works. The buffer contained 25 mM KPi (pH 6.2), 200 mM KCl and 8 mM MgCl2. The spectrum was recorded with 512 scans and 256 points in the second. Carrier frequencies were set to 4.699 ppm (direct dimension) and 7.5 ppm (indirect 1H dimension). The spectral width was set to 25 ppm (direct dimension), and 16 ppm (indirect dimension). Radiofrequency of the hard pulses were set to 11.85 kHz (^{1}H). The hard pulse for ^{15}N decoupling was set to 7.25 kHz. The relaxation delay was set to 1.8 s and the acquisition time was set to 80 ms. Garp4 was used as a decoupling scheme during acquisition. (C) Strip plot of a 3D-[^{1}H,^{15}N,^{1}H]-SOFAST-HMQC-NOESY of the non-modified tRNA^{fMet} construct (440 µM). The buffer contained 25 mM KPi (pH 6.2), 200 mM KCl and 5 mM $MgCl_2$. Each strip has the corresponding chemical shift of the indicated imino resonance. All imino resonances are listed in Supplementary Table S2. The

buffer contained 25 mM KPi (pH 6.2), 200 mM KCl and 5 mM $MgCl_2$. The spectrum was recorded with 96 scans and 60 points and 128 points in the second and third indirect dimension, respectively. Carrier frequencies were set to 4.7 ppm (^1H) and 153.75 ppm (^{15}N). The spectral width was set to 25 ppm (^1H dimension), 22.5 ppm (second ^{15}N dimension) and 12.3 ppm (third ^1H dimension). Radiofrequency of the hard pulses were set to 11.49 kHz (^1H) and 6.41 kHz (^{15}N). The relaxation delay was set to 0.3 s and the acquisition time was set to 43 ms. Garp4.p62 was used as decoupling scheme during acquisition.

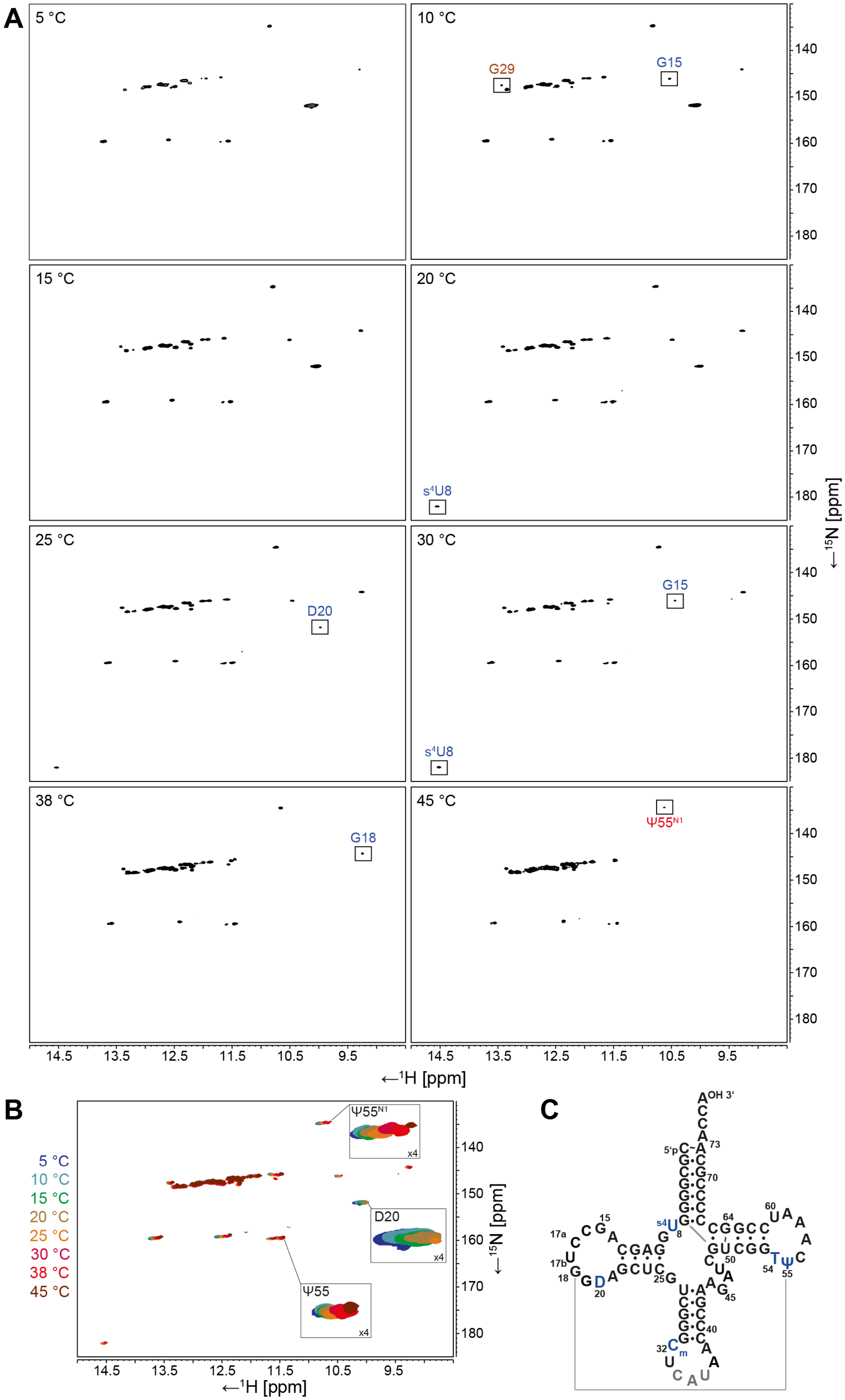
A
5 °C
10 °C
G29
G15
15 °C
20 °C
s4U8
25 °C
D20
30 °C
G15
s4U8
38 °C
G18
45 °C
Ψ55N1
←1H [ppm]
←15N [ppm]
B
5 °C
10 °C
15 °C
20 °C
25 °C
30 °C
38 °C
45 °C
Ψ55N1
D20
Ψ55
x4
C
AOH 3'
5'p
s4U
D
Cm
TΨC

Supplementary Figure S5: Temperature series of 2D-[^{1}H,^{15}N]-BEST-TROSY experiments for the modified tRNA^{fMet} construct (531 µM) acquired at a field strength of either 800 MHz (5 °C to 30 °C) or 600 MHz (38 °C to 45 °C). (A) Temperature series of HN-correlation spectra with highlighted peak (black boxes) which change with temperature. The buffer contained 25 mM KPi (pH 6.2), 200 mM KCl and 5 mM $MgCl_2$. Each spectrum was recorded with either 512 scans (5 °C to 30 °C) or 16 scans (38 °C to 45 °C). Carrier frequencies were set to 4.7 ppm (^{1}H) and 160 ppm (^{15}N). The spectral width was set to 25 ppm (^{1}H dimension) and 60 ppm (15N dimension). Radiofrequency of the hard pulses for protons were set to 15.85 kHz (5 °C), 15.66 kHz (10 °C), 15.48 kHz (15 °C), 15.15 kHz (20 °C), 14.84 kHz (25 °C), 14.32 kHz (30 °C), 16.56 kHz (38 °C), and 15.95 kHz (45 °C). The ^{15}N hard pulse for was set to either 6.58 kHz (5 °C to 30 °C) or 5.95 kHz (38 °C to 45 °C) (^{15}N). The relaxation delay was set to 0.3 s and the acquisition time was set to either 51.2 ms (5 °C to 30 °C) or 80 ms (38 °C to 45 °C). Garp4 was used as decoupling scheme during acquisition. (B) Overlay of spectra shown in (A) with color indication. (C) Secondary structure of modified tRNA^{fMet}.

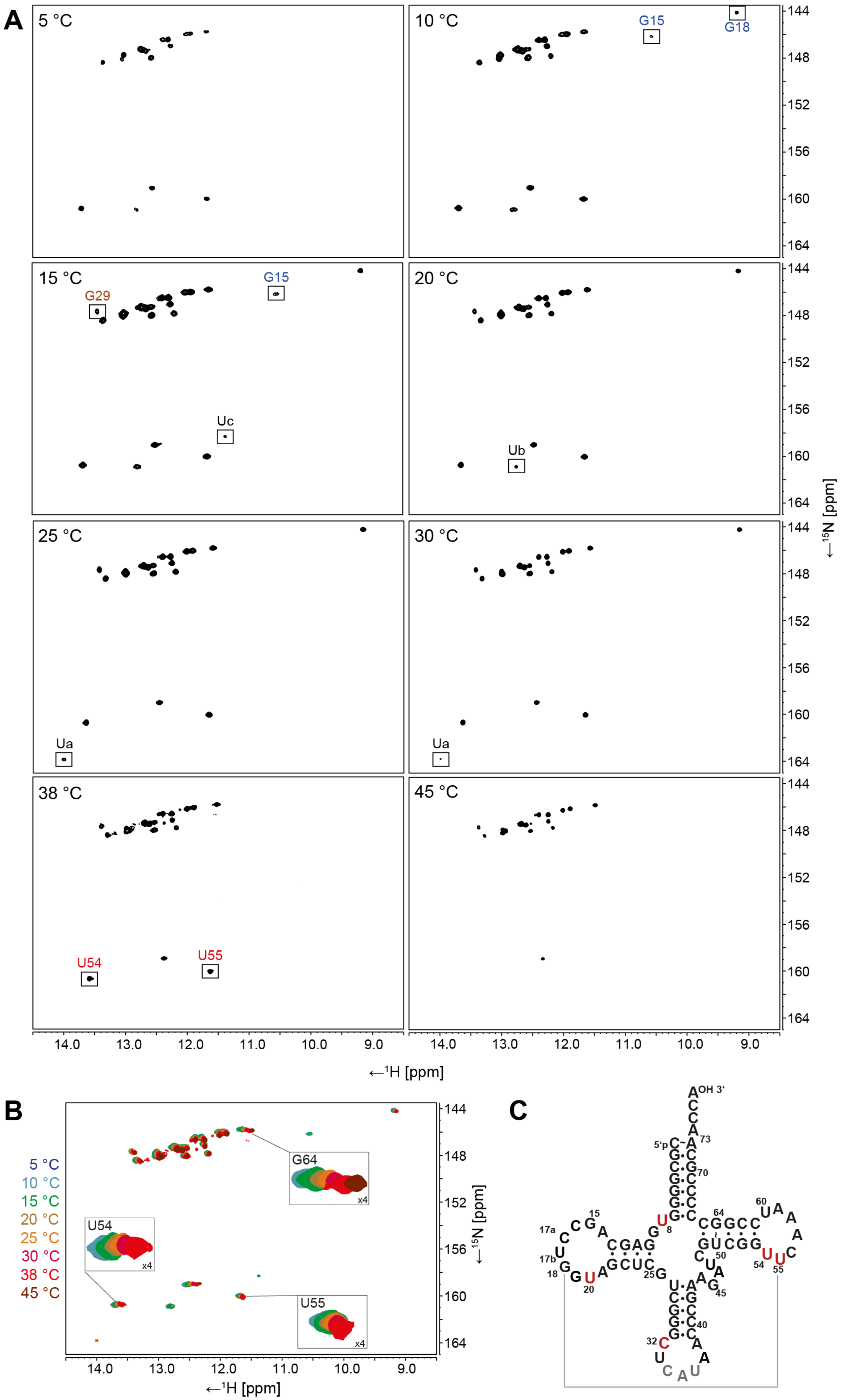
A
5 °C
10 °C
G15
G18
15 °C
G29
G15
Uc
20 °C
Ub
25 °C
Ua
30 °C
Ua
38 °C
U54
U55
45 °C
144
148
152
156
160
164
14.0
13.0
12.0
11.0
10.0
9.0
←¹H [ppm]
←¹⁵N [ppm]
B
5 °C
10 °C
15 °C
20 °C
25 °C
30 °C
38 °C
45 °C
G64
U54
U55
x4
←¹H [ppm]
←¹⁵N [ppm]
C
OH 3'
5'p
73
70
64
60
54
55
50
45
40
32
25
20
18
17a
17b
15
8

Supplementary Figure S6: Temperature series of 2D-[^{1}H,^{15}N]-BEST-TROSY experiments acquired at a field strength of 700 MHz and 600 MHz. (A) Temperature series of HN-correlation spectra with highlighted peak (black boxes) which change with temperature. The buffer contained 25 mM KPi (pH 6.2), 200 mM KCl and 5 mM $MgCl_2$. The spectra acquired at a field strength of 700 MHz and temperatures of up to 30 °C were recorded with 64 scans and 256 points in the indirect ^{15}N dimension. The spectra obtained at a field strength of 600 MHz and temperatures of 38 °C and 45 °C were acquired with 24 scans and 256 points in the indirect dimension. Carrier frequencies were set to 4.7 ppm (^{1}H) and 153.8 ppm (^{15}N). The spectral width was set to 25 ppm (^{1}H dimension) and 22.8 ppm (^{15}N dimension). Radiofrequency of the hard pulses for protons were set to 16.97 kHz (5 °C), 16.32 kHz (10 °C), 15.71 kHz (15 °C), 15.13 kHz (20 °C), 14.59 kHz (25 °C), 14.24 kHz (30 °C), 14.97 kHz (38 °C), and 14.33 kHz (45 °C). The 15N hard pulse was set to either 7.14 kHz (5 °C to 30 °C) or 6.10 kHz (38 °C and 45 °C). The relaxation delay was set to 0.3 s and the acquisition time was set to 58.6 ms. Garp4 was used as decoupling scheme during acquisition. (B) Overlay of spectra shown in (A) with color indication. (C) Secondary structure of non-modified tRNA^{fMet}.

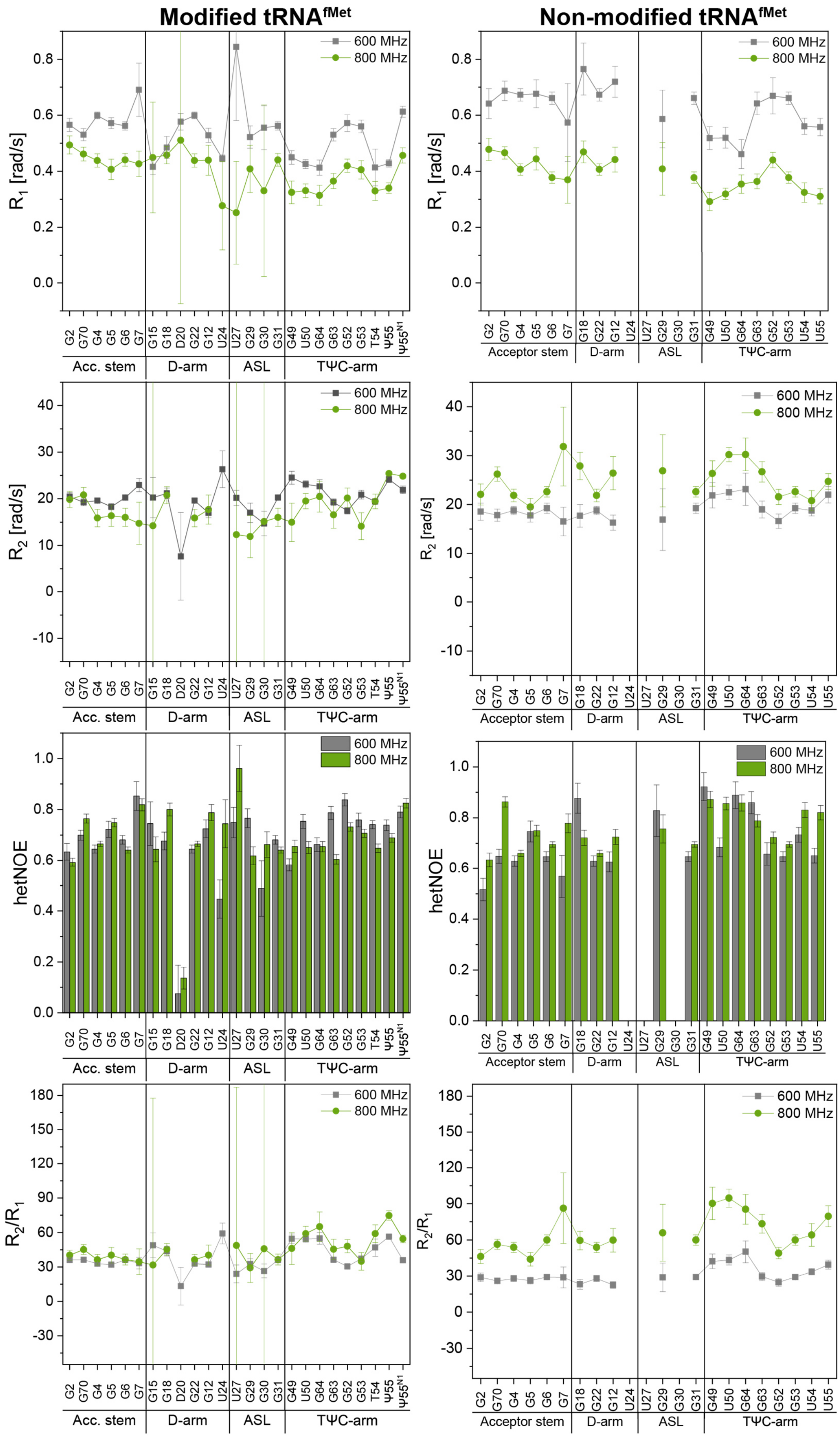

Supplementary Figure S7: Overview of the determined ^{15}N relaxation parameters for the modified and the non-modified tRNA^{fMet} construct at a field strength of 600 MHz (grey) and 800 MHz (green) and 25 °C. The relaxation parameters were determined using pseudo-3D-[^{1}H,^{15}N]-HSQC experiments as described in the method section. The values are listed in Supplementary Table S4 and Supplementary Table S6.

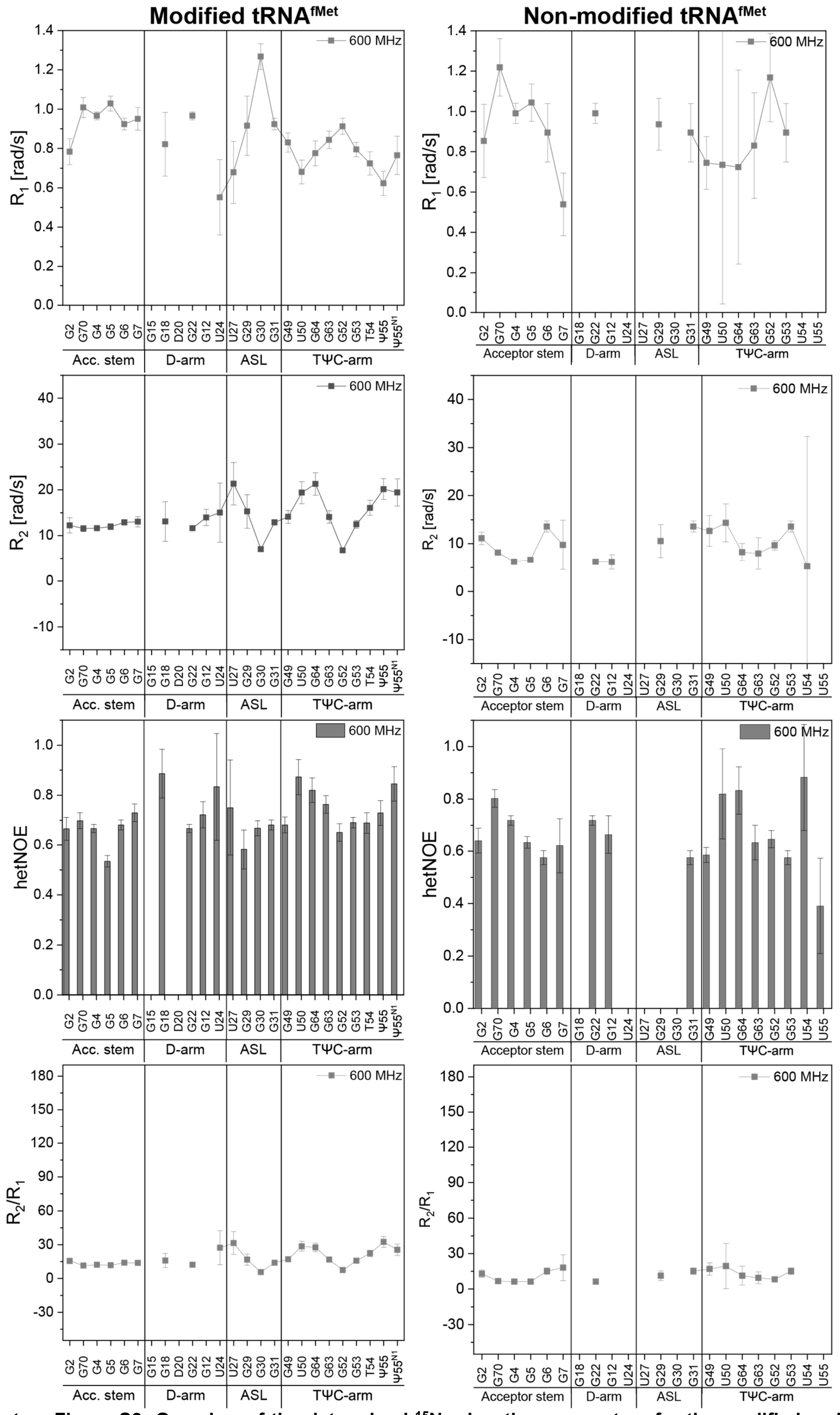

Supplementary Figure S8: Overview of the determined ^{15}N relaxation parameters for the modified and the non-modified tRNA^{fMet} construct at a field strength of 600 MHz (grey) and 40 °C. The relaxation parameters were

determined using pseudo-3D-[^{1}H,^{15}N]-HSQC experiments as described in the method section. The values are listed in Supplementary Table S5 and Supplementary Table S7.

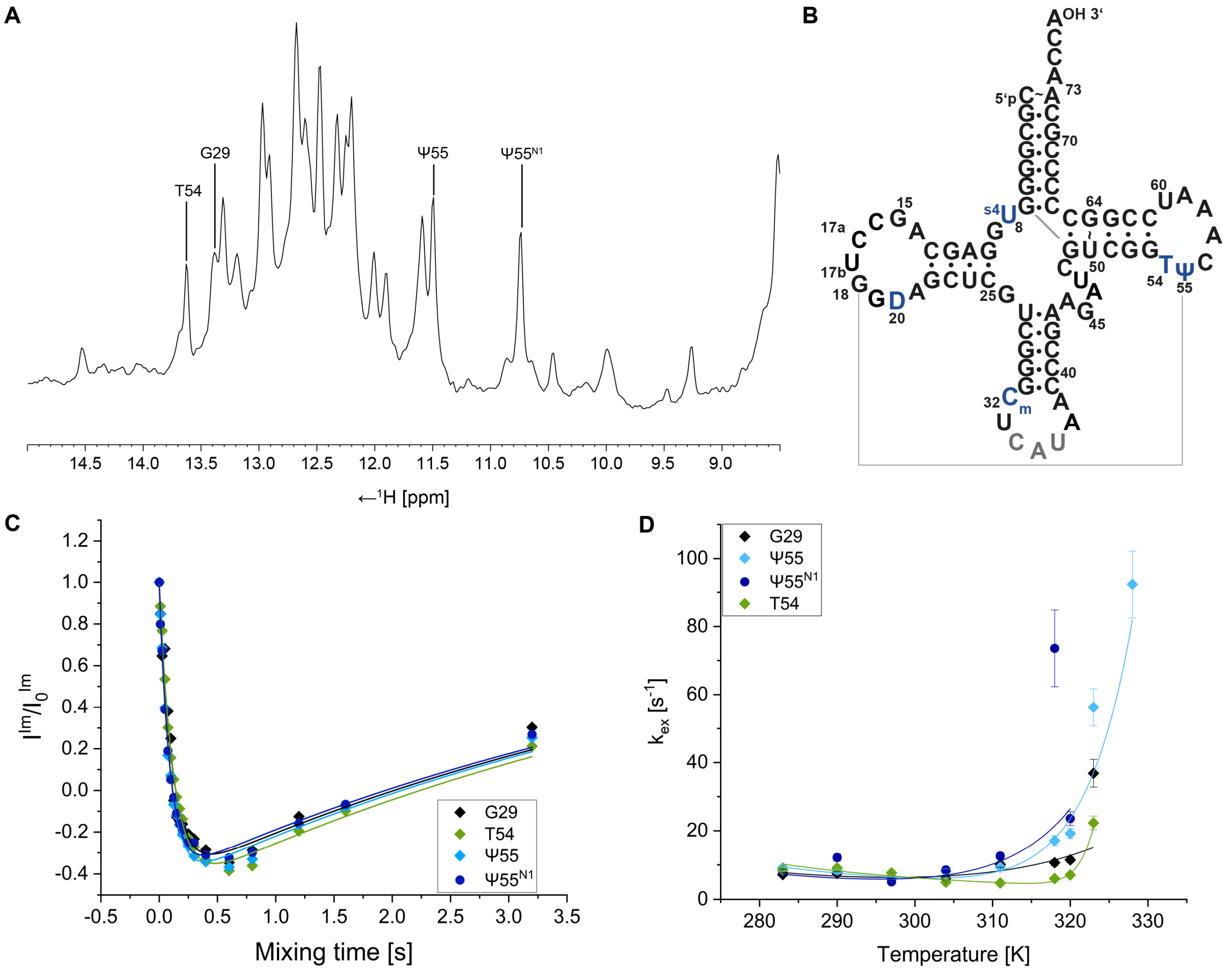

Supplementary Figure S9: Analysis of the water-exchange modulated peak intensities of the modified tRNA^{fMet} construct (440 µM). (A) 1D-jump return proton spectrum recorded with a mixing time τ_M of 1 ms at 297 K. (B) Secondary structure of modified tRNA^{fMet}. (C & D) Exemplary analysis of the τM-modulated peak intensities (I_{Imino}) of the nucleotides G29, T54, and Ψ55. The peak intensities were divided by the peak intensity at τ_M = 1 ms (I_0) and plotted against the corresponding mixing time τ_M. From this the resulting k_{ex} rates in Supplementary Table S13 were then plotted against the temperature. Fitting of this data yielded the thermodynamics properties of each base paired nucleotide as summarized in Supplementary Table S15.

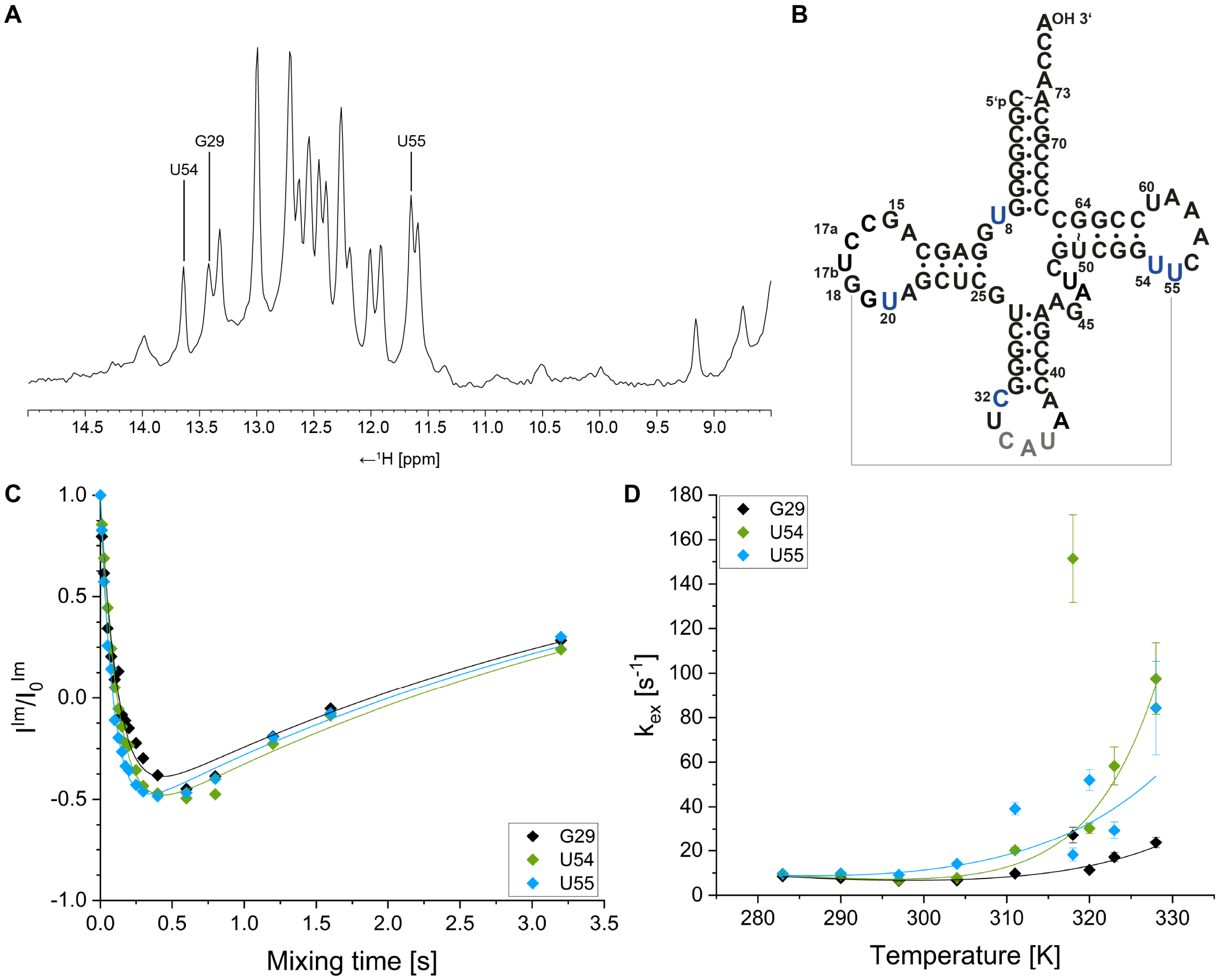

Supplementary Figure S10: Analysis of the water-exchange modulated peak intensities of the non-modified tRNA^{fMet} construct (440 µM). (A) 1D-jump return proton spectrum recorded with a mixing time τ_M of 1 ms at 297 K. (B) Secondary structure of non-modified tRNA^{fMet}. (C & D) Exemplary analysis of the τM-modulated peak intensities (I_{Imino}) of the nucleotides G29, U54, an U55. The peak intensities were divided by the peak intensity at τ_M = 1 ms (I_0) and plotted against the corresponding mixing time τ_M. From this the resulting k_{ex} rates in Supplementary Table S 14 were then plotted against the temperature. Fitting of this data yielded the thermodynamics properties of each base paired nucleotide as summarized in Supplementary Table S16.

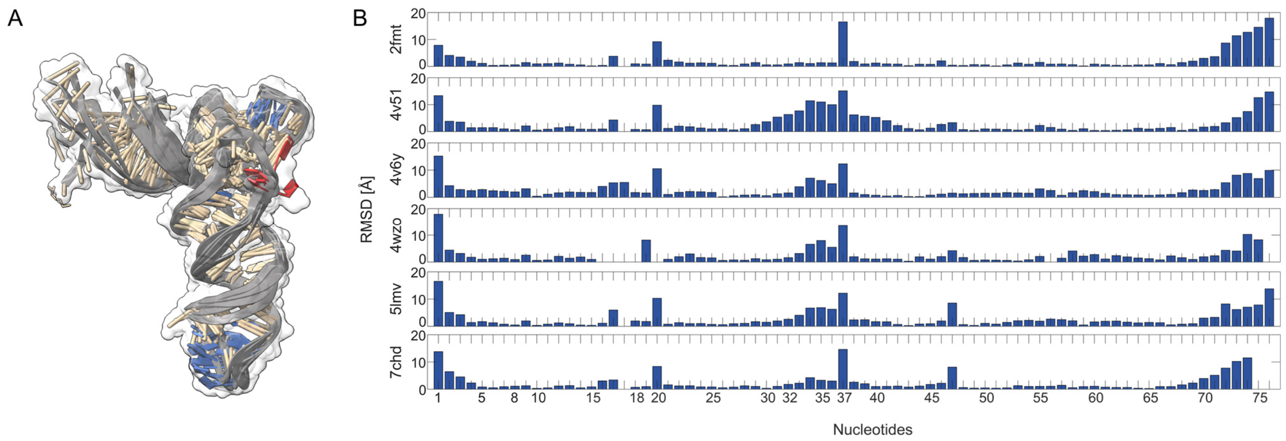

Supplementary Figure S11: Analysis of structural differences between reported crystal structures. (A) Bundle of seven crystal structures, which were aligned to the backbone of the crystal structure of free $tRNA^{fMet}$ (PDB: 3cw6). The nucleobases of the modified nucleotides as well as the anticodon are highlighted in blue. The nucleobase of dihydrouridine 20 is highlighted in red. The remaining six crystal structures of tRNA were isolated from different complexes (PDB: 2fmt, 4v51, 4v6y, 4wzo, 5lmv, 7chd) (B) Root mean square displacement (RMSD) analysis of the aligned tRNA bundle shown in (A). The RSMD values were aligned to the backbone of free $tRNA^{fMet}$ (PDB: 3cw6) and calculated between the average distance of the N1 and N3 atom of the nucleobase. Analysis was performed using ChimeraX[4,5].

Supplementary Table S1: Chemical shifts of the imino resonances for the modified tRNA^{fMet}. The assignment was conducted using the software SPARKY[6] and the experiments shown in Fig. 2 and Supplementary Figure S3. 'n. a.' corresponds to 'not assigned'.

Acc. stem	^{1}H [ppm]	^{15}N [ppm]
G2	12.47	147.7
G70	12.26	146.6
G4	12.70	147.3
G5	12.61	147.4
G6	12.98	148.0
G7	12.56	147.2

ACSL	^{1}H [ppm]	^{15}N [ppm]
G26	n. a.	n. a.
U27	13.69	159.4
G42	n. a.	n. a.
G29	13.40	147.5
G30	13.18	148.3
G31	12.98	148.0
G42	11.90	146.1

D-arm	^{1}H [ppm]	^{15}N [ppm]
s^{4}U8	14.53	182.0
G10	n. a.	n. a.
U24	11.63	159.4
G12	12.00	146.1
G22	12.70	147.3
G15	10.47	146.1
G18	9.29	144.2
G19*	12.21	148.0

TΨC-arm	^{1}H [ppm]	^{15}N [ppm]
G49	13.30	148.4
U50	12.49	146.2
G64	11.60	145.7
G63	12.21	146.9
G52	12.33	146.5
G53	12.92	147.8
T54	13.66	159.3
Ψ55-N3	11.47	159.4
Ψ55-N1	10.70	134.5

Supplementary Table S2: Chemical shifts of the imino resonances for the non-modified tRNA^{fMet} construct. The assignment was conducted using the software SPARKY[6] and the experiments shown in Fig. 3 and Supplementary Figure S4. 'n. a.' corresponds to 'not assigned'.

Acc. stem	^{1}H [ppm]	^{15}N [ppm]
G2	12.53	147.9
G70	12.26	146.5
G4	12.70	147.4
G5	12.63	147.4
G6	12.97	147.9
G7	12.55	147.5

ACSL	^{1}H [ppm]	^{15}N [ppm]
G26	n. a.	n. a.
U27	n. a.	n. a.
G42	n. a.	n. a.
G29	13.42	147.6
G30	n. a.	n. a.
G31	13.00	147.9

D-arm	^{1}H [ppm]	^{15}N [ppm]
U8	14.22	162.4
G10	n. a.	n. a.
U24	n. a.	n. a.
G12	11.98	146.1
G22	12.72	147.4
G15	10.52	146.2
G18	9.16	144.2
G19	n. a.	n. a.

TΨC-arm	^{1}H [ppm]	^{15}N [ppm]
G49	13.32	148.4
U50	12.45	158.9
G64	11.58	145.8
G63	12.24	147.0
G52	12.39	146.5
G53	13.00	147.9
U54	13.63	160.7
U55	11.64	160.0

Supplementary Table S3: Overview of total, base-paired and assigned guanidine and uridine nucleotides in non-modified and modified tRNA. The corresponding chemical shifts are listed in Supplementary Table S1 and Supplementary Table S2.

		Total Nucleotides	Thereof base-paired	Thereof assigned	Percentage assigned
Non-modified tRNA	G	24	22	17	77
	U	12	6	4	67
	G & U	36	28	21	72
Modified tRNA	G	24	22	20	91
	U	12	7	7	100
	G & U	36	29	26	96

Supplementary Table S4: ^{15}N relaxation rates of the imino groups in modified $tRNA^{fMet}$ at 25 °C. The relaxation rates were determined as described in the methods section. Analysis of the spectra were conducted with the software DynamicsCenter from Bruker Biospin.

Nucleotide		R_1 [rad/s]		R_2 [rad/s]		$hetNOE$	
		600 MHz	800 MHz	600 MHz	800 MHz	600 MHz	800 MHz
Acceptor stem	G2	0.57 ± 0.02	0.49 ± 0.03	20.4 ± 0.9	19.9 ± 1.7	0.63 ± 0.03	0.59 ± 0.02
	G70	0.53 ± 0.02	0.46 ± 0.02	19.2 ± 0.7	20.8 ± 1.6	0.7 ± 0.02	0.76 ± 0.02
	G4	0.599 ± 0.012	0.44 ± 0.02	19.6 ± 0.5	15.9 ± 1.9	0.64 ± 0.02	0.67 ± 0.01
	G5	0.57 ± 0.02	0.41 ± 0.04	18.3 ± 0.7	16.4 ± 2.3	0.72 ± 0.03	0.75 ± 0.02
	G6	0.56 ± 0.02	0.44 ± 0.02	20.2 ± 0.6	16 ± 1.9	0.68 ± 0.02	0.64 ± 0.01
	G7	0.69 ± 0.1	0.43 ± 0.05	22.9 ± 1.4	14.7 ± 4.5	0.85 ± 0.06	0.82 ± 0.02
D-arm	G15	0.42 ± 0.03	0.45 ± 0.2	20.3 ± 4.3	14.2 ± 65.3	0.74 ± 0.09	0.64 ± 0.05
	G18	0.48 ± 0.04	0.46 ± 0.03	21.2 ± 1.1	20.8 ± 1.8	0.68 ± 0.03	0.8 ± 0.03
	D20	0.58 ± 0.03	0.51 ± 0.58	7.6 ± 9.4	n. d.	0.07 ± 0.11	0.14 ± 0.04
	G22	0.6 ± 0.01	0.44 ± 0.02	19.6 ± 0.5	15.9 ± 1.9	0.64 ± 0.02	0.67 ± 0.01
	G12	0.53 ± 0.02	0.44 ± 0.05	16.9 ± 1	17.7 ± 3.1	0.72 ± 0.04	0.79 ± 0.03
	U24	0.446 ± 0.013	0.28 ± 0.16	26.3 ± 3.9	n. d.	0.45 ± 0.08	0.74 ± 0.09
ACSL	U27	0.84 ± 0.26	0.25 ± 0.18	20.2 ± 1.6	12 ± 34	0.75 ± 0.06	0.96 ± 0.09
	G29	0.52 ± 0.04	0.41 ± 0.08	17 ± 2.1	12 ± 5	0.77 ± 0.04	0.62 ± 0.04
	G30	0.56 ± 0.08	0.33 ± 0.31	14.7 ± 2.7	15 ± 118	0.49 ± 0.11	0.66 ± 0.05
	G31	0.56 ± 0.02	0.44 ± 0.02	20.2 ± 0.6	16 ± 1.9	0.68 ± 0.02	0.64 ± 0.01
TΨC-arm	G49	0.45 ± 0.02	0.32 ± 0.04	24.5 ± 1.4	14.9 ± 4.1	0.58 ± 0.02	0.65 ± 0.02
	U50	0.43 ± 0.02	0.33 ± 0.02	23.1 ± 0.8	19.5 ± 1.6	0.75 ± 0.03	0.65 ± 0.02
	G64	0.41 ± 0.03	0.31 ± 0.04	22.7 ± 1.4	20.4 ± 3.3	0.66 ± 0.03	0.65 ± 0.02
	G63	0.53 ± 0.02	0.36 ± 0.03	19.2 ± 0.7	16.5 ± 2.9	0.79 ± 0.03	0.6 ± 0.02
	G52	0.57 ± 0.03	0.42 ± 0.02	17.4 ± 0.7	20.2 ± 2.1	0.84 ± 0.02	0.73 ± 0.02
	G53	0.56 ± 0.02	0.41 ± 0.03	20.9 ± 0.9	14.1 ± 2.9	0.76 ± 0.03	0.71 ± 0.02
	T54	0.41 ± 0.07	0.33 ± 0.03	19.4 ± 0.5	19.4 ± 1.6	0.74 ± 0.02	0.65 ± 0.02
	Ψ55	0.428 ± 0.014	0.34 ± 0.02	24.1 ± 0.7	25.41 ± 0.03	0.74 ± 0.02	0.69 ± 0.02
	$Ψ55^{N1}$	0.61 ± 0.02	0.46 ± 0.03	21.9 ± 0.8	24.8 ± 0.4	0.79 ± 0.02	0.83 ± 0.02

Supplementary Table S5: ^{15}N relaxation rates of the imino groups in modified $tRNA^{fMet}$ at 40 °C. The relaxation rates were determined as described in the methods section. Analysis of the spectra were conducted with the software DynamicsCenter from Bruker Biospin.

Nucleotide		R_1 [rad/s]	R_2 [rad/s]	$hetNOE$
		600 MHz	600 MHz	600 MHz
Acceptor stem	G2	0.78 ± 0.07	12.2 ± 1.7	0.67 ± 0.05
	G70	1.01 ± 0.05	11.5 ± 0.7	0.7 ± 0.03
	G4	0.97 ± 0.02	11.6 ± 0.4	0.67 ± 0.02
	G5	1.03 ± 0.04	12 ± 0.6	0.53 ± 0.02
	G6	0.92 ± 0.03	12.9 ± 0.6	0.68 ± 0.02
	G7	0.95 ± 0.06	13 ± 1.1	0.73 ± 0.04
D-arm	G15	n. d.	n. d.	n. d.
	G18	0.82 ± 0.16	13 ± 4	0.89 ± 0.1
	D20	n. d.	n. d.	n. d.
	G22	0.97 ± 0.02	11.6 ± 0.4	0.67 ± 0.02
	G12	n. d.	14 ± 1.8	0.72 ± 0.05
	U24	0.55 ± 0.19	15 ± 6	0.8 ± 0.2
ACSL	U27	0.68 ± 0.16	21 ± 5	0.8 ± 0.2
	G29	0.92 ± 0.15	15 ± 4	0.58 ± 0.08
	G30	1.27 ± 0.06	7.0 ± 0.5	0.67 ± 0.03
	G31	0.92 ± 0.03	12.9 ± 0.6	0.68 ± 0.02
TΨC-arm	G49	0.83 ± 0.05	14.1 ± 1.4	0.68 ± 0.03
	U50	0.68 ± 0.06	19 ± 2	0.87 ± 0.07
	G64	0.78 ± 0.06	21 ± 2	0.82 ± 0.05
	G63	0.84 ± 0.04	14.1 ± 1.3	0.76 ± 0.03
	G52	0.91 ± 0.04	6.8 ± 0.4	0.65 ± 0.03
	G53	0.79 ± 0.04	12.5 ± 0.9	0.69 ± 0.02
	T54	0.72 ± 0.06	16 ± 1.6	0.69 ± 0.04
	Ψ55	0.62 ± 0.06	20 ± 2	0.73 ± 0.05
	$Ψ55^{N1}$	0.77 ± 0.1	19 ± 3	0.85 ± 0.07

Supplementary Table S6: ^{15}N relaxation rates of the imino groups in non-modified tRNA^{fMet} at 25 °C. The relaxation rates were determined as described in the methods section. Analysis of the spectra were conducted with the software DynamicsCenter from Bruker Biospin.

Nucleotide		R_1 [rad/s]		R_2 [rad/s]		$hetNOE$	
		600 MHz	800 MHz	600 MHz	800 MHz	600 MHz	800 MHz
Acceptor stem	G2	0.64 ± 0.05	0.48 ± 0.04	18.5 ± 1.8	22.1 ± 2.1	0.52 ± 0.04	0.63 ± 0.03
	G70	0.69 ± 0.03	0.47 ± 0.02	17.8 ± 1.3	26.2 ± 1.5	0.65 ± 0.03	0.86 ± 0.02
	G4	0.67 ± 0.02	0.41 ± 0.02	18.8 ± 0.9	21.9 ± 1.3	0.63 ± 0.02	0.661 ± 0.012
	G5	0.68 ± 0.05	0.44 ± 0.04	17.8 ± 1.3	19.5 ± 1.8	0.75 ± 0.04	0.75 ± 0.02
	G6	0.66 ± 0.02	0.38 ± 0.02	19.3 ± 1	22.6 ± 1.1	0.65 ± 0.02	0.695 ± 0.01
	G7	0.57 ± 0.14	0.37 ± 0.08	17 ± 3	32 ± 8	0.57 ± 0.08	0.78 ± 0.04
D-arm	G15	0.76 ± 0.09	0.47 ± 0.04	18 ± 2	28 ± 3	0.88 ± 0.06	0.72 ± 0.03
	G18	0.67 ± 0.02	0.41 ± 0.02	18.8 ± 0.9	21.9 ± 1.3	0.63 ± 0.02	0.66 ± 0.01
	G22	0.72 ± 0.06	0.44 ± 0.04	16.3 ± 1.6	26.4 ± 3.4	0.63 ± 0.04	0.72 ± 0.03
	G12	n. d.	n. d.	n. d.	n. d.	n. d.	n. d.
	U24	n. d.	n. d.	n. d.	n. d.	n. d.	n. d.
ACSL	U27	0.59 ± 0.1	0.41 ± 0.09	16.9 ± 6.3	26.9 ± 7.4	0.83 ± 0.1	0.76 ± 0.06
	G29	n. d.	n. d.	n. d.	n. d.	n. d.	n. d.
	G30	0.66 ± 0.02	0.38 ± 0.02	19.3 ± 1	22.6 ± 1.1	0.65 ± 0.02	0.695 ± 0.01
	G31	0.52 ± 0.04	0.29 ± 0.03	22 ± 3	26 ± 3	0.92 ± 0.06	0.87 ± 0.03
TΨC-arm	G49	0.52 ± 0.04	0.32 ± 0.02	22.5 ± 1.5	30.2 ± 1.5	0.68 ± 0.04	0.86 ± 0.03
	U50	0.46 ± 0.05	0.35 ± 0.03	23 ± 3	30 ± 3	0.89 ± 0.05	0.86 ± 0.03
	G64	0.64 ± 0.04	0.36 ± 0.03	19 ± 1.7	26.7 ± 2.1	0.86 ± 0.04	0.79 ± 0.02
	G63	0.67 ± 0.06	0.44 ± 0.03	16.6 ± 1.5	21.6 ± 1.5	0.66 ± 0.04	0.72 ± 0.02
	G52	0.66 ± 0.02	0.38 ± 0.02	19.3 ± 1	22.6 ± 1.1	0.65 ± 0.02	0.695 ± 0.01
	G53	0.56 ± 0.03	0.32 ± 0.04	18.8 ± 1.1	20.8 ± 2.1	0.73 ± 0.03	0.83 ± 0.03
	U54	0.56 ± 0.03	0.31 ± 0.03	22 ± 1.7	24.7 ± 1.6	0.65 ± 0.03	0.82 ± 0.03
	U55	0.64 ± 0.05	0.48 ± 0.04	18.5 ± 1.8	22.1 ± 2.1	0.52 ± 0.04	0.63 ± 0.03

Supplementary Table S7: ^{15}N relaxation rates of the imino groups in non-modified $tRNA^{fMet}$ at 40 °C. The relaxation rates were determined as described in the methods section. Analysis of the spectra were conducted with the software DynamicsCenter from Bruker Biospin.

Nucleotide		R_1 [rad/s]	R_2 [rad/s]	$hetNOE$
		600 MHz	600 MHz	600 MHz
Acceptor stem	G2	0.9 ± 0.2	11.1 ± 1.3	0.64 ± 0.05
	G70	1.22 ± 0.14	8.1 ± 0.4	0.8 ± 0.03
	G4	0.99 ± 0.05	6.2 ± 0.3	0.72 ± 0.02
	G5	1.04 ± 0.09	6.6 ± 0.4	0.63 ± 0.02
	G6	0.89 ± 0.14	13.6 ± 1.2	0.58 ± 0.03
	G7	0.5 ± 0.2	10 ± 5	0.62 ± 0.1
	G18	n. d.	n. d.	n. d.
	G22	0.99 ± 0.05	6.2 ± 0.3	0.72 ± 0.02
	G12	n. d.	6.2 ± 1.5	0.66 ± 0.07
	U24	n. d.	n. d.	n. d.
ACSL	U27	n. d.	n. d.	n. d.
	G29	0.94 ± 0.13	11 ± 3	n. d.
	G30	n. d.	n. d.	n. d.
	G31	0.89 ± 0.14	13.6 ± 1.2	0.58 ± 0.03
TΨC-arm	G49	0.74 ± 0.13	13 ± 3	0.59 ± 0.03
	U50	0.7 ± 0.7	14 ± 4	0.82 ± 0.17
	G64	0.7 ± 0.5	8.2 ± 1.8	0.83 ± 0.09
	G63	0.8 ± 0.3	8 ± 3	0.63 ± 0.07
	G52	1.2 ± 0.2	9.7 ± 1	0.65 ± 0.03
	G53	0.89 ± 0.14	13.6 ± 1.2	0.58 ± 0.03
	U54	n. d.	5 ± 27	0.88 ± 0.2
	U55	n. d.	n. d.	0.39 ± 0.18

Supplementary Table S8: Motion models used in the model-free analysis as proposed by d'Auvergne and Gooley[7,8].

M0	-	$J(\omega) = \frac{2}{5}\sum_{i=-k}^{k} c_\mathrm{i} \cdot \tau_\mathrm{i}\left(\frac{S^2}{1+(\omega\tau_\mathrm{i})^2} + \frac{(1-S_\mathrm{f}^2)(\tau_\mathrm{f}+\tau_\mathrm{i})\tau_\mathrm{f}}{(\tau_\mathrm{f}+\tau_\mathrm{i})^2+(\omega\tau_\mathrm{f}\tau_\mathrm{i})^2} + \frac{(S_\mathrm{f}^2-S^2)(\tau_\mathrm{s}+\tau_\mathrm{i})\tau_\mathrm{s}}{(\tau_\mathrm{s}+\tau_\mathrm{i})^2+(\omega\tau_\mathrm{s}\tau_\mathrm{i})^2}\right)$
M1	S^2	
M2	S^2, τ_e	
M3	S^2, R_ex	
M4	$S^2, \tau_\mathrm{e}, R_\mathrm{ex}$	
M5	$S^2, S_\mathrm{f}^2, \tau_\mathrm{s}$	
M6	$S^2, \tau_\mathrm{f}, S_\mathrm{f}^2, \tau_\mathrm{s}$	
M7	$S^2, S_\mathrm{f}^2, \tau_\mathrm{s}, R_\mathrm{ex}$	
M8	$S^2, \tau_\mathrm{f}, S_\mathrm{f}^2, \tau_\mathrm{s}, R_\mathrm{ex}$	
M9	R_ex	

Supplementary Table S9: Determined structural dynamic parameters of the individual imino groups for the modified tRNA^{fMet} at 25 °C. The parameters were determined using the obtained ^{15}N relaxation parameters of Supplementary Table S4, which were analyzed with the Model-free approach. Models are given in Supplementary Table S8. The R_{ex} value is given at a magnetic field of 600 MHz. 'n. d.' corresponds to 'not detectable'.

Nt.		Model	S^2	S_f^2	τ_f [ps]	τ_s [ps]	R_{ex} [s^{-1}]
Acc. stem	G2	M8	0.77 ± 0.10	0.88 ± 0.07	40 ± 30	3000 ± 3000	0 ± 2
	G70	M6	0.688±0.07	0.90 ± 0.03	60 ± 40	12000 ± 7000	-
	G4	M6	0.89 ± 0.02	0.96 ± 0.02	60 ± 80	1700 ± 1500	-
	G5	M2	0.97 ± 0.02	-	60 ± 180	-	-
	G6	M4	0.96 ± 0.01	-	-	130 ± 70	1.0 ± 0.5
	G7	M2	0.91 ± 0.07	-	-	5000 ± 5000	
D-arm	G15	M4	0.66 ± 0.04	-	7 ± 2		8 ± 4
	G18	M7	0.8 ± 0.2	0.85 ± 0.13		1500 ± 900	4 ± 2
	D20	M6	0.52 ± 0.12	0.67 ± 0.11	30 ± 20	1000 ± 2000	-
	G22	M6	0.78 ± 0.02	0.89 ± 0.02	24 ± 6	2500 ± 600	-
	G12	M5	0.66 ± 0.04	0.77 ± 0.03	-	2300 ± 600	-
	U24	M5	0.76 ± 0.09	0.80 ± 0.10	-	600 ± 400	-
ACSL	U27	M1	0.83 ± 0.06	-	-	-	-
	G29	M2	0.87 ± 0.05	-	20 ± 90	-	-
	G30	n. d.	n. d.	n. d.	n. d.	-	-
	G31	M4	0.96 ± 0.01	-	-	120 ± 70	1.1 ± 0.6
TΨC-arm	G49	M5	0.90 ± 0.04	0.94 ± 0.04	-	600 ± 200	-
	U50	M2	0.891±0.020	-	19 ± 5	-	-
	G64	M2	0.938±0.015	-	-	1300 ± 200	-
	G63	M2	0.971±0.017	-	-	110 ± 150	-
	G52	M2	0.968±0.020	-	50 ± 190	-	-
	G53	M2	0.954±0.021	-	90 ± 90	-	-
	T54	M2	0.838±0.021	-	13 ± 2	-	-
	Ψ55-N3	M2	0.903±0.001	-	18 ± 2	-	-
	Ψ55-N1	M2	0.965±0.017	-	-	3000 ± 4000	-

Supplementary Table S10: Determined structural dynamic parameters of the individual imino groups for the modified tRNA^{fMet} at 40 °C. The parameters were determined using the obtained ^{15}N relaxation parameters of Supplementary Table S5, which were analyzed with the Model-free approach. Models are given in Supplementary Table S8. The R_{ex} value is given at a magnetic field of 600 MHz. 'n. d.' corresponds to 'not detectable'.

Nt.		Model	S^2	S_f^2	τ_f [ps]	τ_s [ps]	R_{ex} [s^{-1}]
Acc. stem	G2	M2	0.94 ± 0.05	-	100 ± 400	-	-
	G70	M3	0.93 ± 0.05	-	-	-	0.0 ± 0.7
	G4	M2	0.84 ± 0.01	-	-	1680 ± 130	-
	G5	M2	0.86 ± 0.03	-	-	1600 ± 300	-
	G6	M2	0.94 ± 0.02	-	-	900 ± 400	-
	G7	M2	0.81 ± 0.04	-	-	1200 ± 200	-
D-arm	G15	n. d.	n. d.	n. d.	n. d.	n. d.	n. d.
	G18	M0	-	-	-	-	
	D20	n. d.	n. d.	n. d.	n. d.	n. d.	n. d.
	G22	M2	0.79 ± 0.01	-	-	1940 ± 130	-
	G12	n. d.	n. d.	n. d.	n. d.	n. d.	n. d.
	U24	n. d.	n. d.	n. d.	n. d.	n. d.	n. d.
ACSL	U27	M0	-	-	-	-	-
	G29	n. d.	n. d.	n. d.	n. d.	n. d.	n. d.
	G30	M2	0.57 ± 0.04	-	-	1700 ± 300	-
	G31	M2	0.94 ± 0.02	-	-	800 ± 400	-
TΨC-arm	G49	M2	0.89 ± 0.03	-	-	1500 ± 400	-
	U50	M9	-	-	-	-	-
	G64	M9	-	-	-	-	6 ± 3
	G63	M0	-	-	-	-	-
	G52	M2	0.72 ± 0.03	-	22 ± 5	-	-
	G53	M2	0.94 ± 0.03	-	100 ± 200	-	-
	T54	M2	0.94 ± 0.04	-	100 ± 400	-	-
	Ψ55-N3	M2	0.93 ± 0.06	-	0 ± 600	-	-
	Ψ55-N1	M9	-	-	-	-	6 ± 3

Supplementary Table S11: Determined structural dynamic parameters of the individual imino groups for the non-modified tRNA^{fMet} at 25 °C. The parameters were determined using the obtained ^{15}N relaxation parameters of Supplementary Table S6, which were analyzed with the Model-free approach. Models are given in Supplementary Table S8. The R_{ex} value is given at a magnetic field of 600 MHz. 'n. d.' corresponds to 'not detectable'.

Nt.		Model	S^2	S_f^2	τ_f [ps]	τ_s [ps]	R_{ex} [s^{-1}]
Acc. stem	G2	M2	0.87 ± 0.02	-	-	1070 ± 110	-
	G70	M7	0.3 ± 0.2	0.56 ± 0.14	-	3700 ± 800	10 ± 3
	G4	M4	0.95 ± 0.01	-	-	590± 120	1.3 ± 0.6
	G5	M2	0.98 ± 0.02	-	-	300 ± 400	-
	G6	M4	0.97 ± 0.01	-	-	380 ± 150	2.2 ± 0.5
	G7	M2	0.96 ± 0.04	-	-	1000 ± 800	-
D-arm	G15	n. d.	n. d.	n. d.	n. d.	n. d.	n. d.
	G18	M4	0.98 ± 0.04	-	-	100 ± 400	3.6 ± 1.4
	G22	M4	0.95 ± 0.01	-	-	510 ± 130	1.5 ± 0.6
	G12	M4	0.94 ± 0.02	-	-	800 ± 300	2.0 ± 1.2
	U24	n. d.	n. d.	n. d.	n. d.	n. d.	n. d.
ACSL	U27	n. d.	n. d.	n. d.	n. d.	n. d.	n. d.
	G29	M0	-	-	-	-	-
	G30	n. d.	n. d.	n. d.	n. d.	n. d.	n. d.
	G31	M4	0.97 ± 0.01	-	-	410 ± 150	2.1 ± 0.5
TΨC-arm	G49	M0	-	-	-	-	-
	U50	M9	-	-	-	-	2.7 ± 0.7
	G64	M9	-	-	-	-	3.1 ± 1.6
	G63	M9	-	-	-	-	2.3 ± 0.9
	G52	M4	0.96 ± 0.02	-	-	600 ± 300	1.4 ± 0.7
	G53	M4	0.96 ± 0.01	-	-	540 ± 150	1.6 ± 0.6
	U54	M1	0.95 ± 0.03	-	-	-	-
	U55	M2	0.98 ± 0.01	-	-	700 ± 400	-

Supplementary Table S 12: Determined structural dynamic parameters of the individual imino groups for the non-modified tRNA^{fMet} at 40 °C. The parameters were determined using the obtained ^{15}N relaxation parameters of Supplementary Table S7, which were analyzed with the Model-free approach. Models are given in Supplementary Table S8. The R_{ex} value is given at a magnetic field of 600 MHz. 'n. d.' corresponds to 'not detectable'.

Nt.		Model	S^2	S_f^2	τ_f [ps]	τ_s [ps]	R_{ex} [s^{-1}]
Acc. stem	G2	M2	0.88 ± 0.09	-	70 ± 500	-	-
	G70	M2	0.83 ± 0.09	-	-	4000 ± 2000	-
	G4	M2	0.88 ± 0.03	-	60 ± 50	-	-
	G5	M2	0.68 ±0.04	-	21 ± 6	-	-
	G6	M2	0.78 ± 0.07	-	20 ± 70	-	-
	G7	M2	0.65 ± 0.07	-	15 ± 8	-	-
D-arm	G15	n. d.	n. d.	n. d.	n. d.	n. d.	n. d.
	G18	n. d.	n. d.	n. d.	n. d.	n. d.	n. d.
	G22	M2	0.86 ± 0.03	-	50 ± 20	-	-
	G12	M2	0.66 ± 0.09	-	19 ± 16	-	-
	U24	n. d.	n. d.	n. d.	n. d.	n. d.	n. d.
ACSL	U27	n. d.	n. d.	n. d.	n. d.	n. d.	n. d.
	G29	n. d.	n. d.	n. d.	n. d.	n. d.	n. d.
	G30	n. d.	n. d.	n. d.	n. d.	n. d.	n. d.
	G31	M2	0.79 ± 0.07	-	30 ± 90	-	-
TΨC-arm	G49	M2	0.73 ± 0.09	-	20 ± 90	-	-
	U50	M1	0.54 ± 0.16	-	-	-	-
	G64	M1	0.67 ± 0.18	-	-	-	-
	G63	n. d.	n. d.	n. d.	n. d.	n. d.	n. d.
	G52	M2	0.90 ± 0.04	-	60 ± 500	-	-
	G53	M2	0.88 ± 0.06	-	50 ± 400	-	-
	U54	n. d.	n. d.	n. d.	n. d.	n. d.	n. d.
	U55	n. d.	n. d.	n. d.	n. d.	n. d.	n. d.

Supplementary Table S13: Determined water exchange rates (k_{ex}) of the imino protons in the modified tRNA[fMet]. The k_{ex} rates are obtained by fitting the signal intensities of the imino resonances.'n. d.' corresponds to 'not detectable'.

Nt.		k_{ex} [s^{-1}]								
		283 K	290 K	297 K	304 K	311 K	318 K	320 K	323 K	328 K
Acceptor stem	G2	9.0 ± 0.8	8.0 ± 0.6	6.6 ± 0.3	6.2 ± 0.6	7.3 ± 0.5	11.3 ± 1.0	11.3 ± 1.0	33.4 ± 12.6	7.6 ± 1.4
	G70	7.4 ± 0.6	7.3 ± 0.5	6.7 ± 0.4	6.1 ± 0.6	6.6 ± 0.4	4.6 ± 0.4	4.6 ± 0.4	7.9 ± 2.1	28 ± 6
	G4	8.4 ± 0.8	8.1 ± 0.6	7.4 ± 0.4	6.9 ± 0.8	6.4 ± 0.4	5.9 ± 0.5	5.9 ± 0.5	6.2 ± 1.8	7.3 ± 1.2
	G5	8.3 ± 0.8	7.3 ± 0.5	6.6 ± 0.4	5.9 ± 0.7	5.6 ± 0.4	5.4 ± 0.5	5.4 ± 0.5	5.2 ± 1.5	41 ± 10
	G6	8.6 ± 0.8	7.7 ± 0.5	6.7 ± 0.4	6.2 ± 0.6	5.5 ± 0.4	6.3 ± 0.5	6.3 ± 0.5	6.7 ± 2.0	7.6 ± 0.4
	G7	8.3 ± 0.7	7.8 ± 0.5	6.2 ± 0.3	5.2 ± 0.5	4.9 ± 0.3	5.4 ± 0.5	5.4 ± 0.5	5.8 ± 1.6	7.3 ± 1.2
D-arm	G15	n. d.	n. d.	n. d.	n. d.	n. d.	n. d.	n. d.	n. d.	n. d.
	G18	n. d.	n. d.	n. d.	n. d.	n. d.	n. d.	n. d.	n. d.	n. d.
	D20	n. d.	n. d.	n. d.	n. d.	n. d.	n. d.	n. d.	n. d.	n. d.
	G22	8.4 ± 0.8	8.1 ± 0.6	7.4 ± 0.4	6.9 ± 0.8	6.4 ± 0.4	5.9 ± 0.5	5.9 ± 0.5	6.2 ± 1.8	7.3 ± 1.2
	G12	8.3 ± 0.8	8.1 ± 0.6	8.4 ± 0.5	5.5 ± 0.5	8.1 ± 0.5	10.5 ± 0.9	10.5 ± 0.9	8.7 ± 1.2	25 ± 5
ACSL	U27	8.0 ± 0.7	6.8 ± 0.4	5.1 ± 0.3	5.6 ± 0.5	2.2 ± 0.1	2.0 ± 0.2	1.5 ± 0.1	n. d.	n. d.
	G29	7.2 ± 0.6	7.5 ± 0.5	6.3 ± 0.3	6.1 ± 0.6	10.1 ± 0.8	10.6 ± 0.9	10.6 ± 0.9	77.9 ± 13.3	n. d.
	G30	7.8 ± 0.7	8.4 ± 0.6	7.9 ± 0.4	6.9 ± 0.7	7.9 ± 0.5	8.9 ± 0.8	8.9 ± 0.8	34.3 ± 12.5	43 ± 12
	G31	8.6 ± 0.8	7.7 ± 0.5	6.7 ± 0.4	6.2 ± 0.6	5.5 ± 0.4	6.3 ± 0.5	6.3 ± 0.5	6.7 ± 2.0	7.6 ± 0.4
TΨC-arm	G49	7.5 ± 0.7	7.0 ± 0.5	6.4 ± 0.3	7.2 ± 0.8	5.9 ± 0.4	6.7 ± 0.6	6.7 ± 0.6	13.2 ± 4.3	8.6 ± 1.6
	U50	9.0 ± 0.8	8.0 ± 0.6	6.6 ± 0.3	6.2 ± 0.6	7.3 ± 0.5	11.3 ± 1.0	11.3 ± 1.0	33.4 ± 12.6	7.6 ± 1.4
	G64	7.0 ± 0.7	8.3 ± 0.6	7.8 ± 0.4	9.2 ± 1.0	7.5 ± 0.5	9.9 ± 0.9	9.9 ± 0.9	49.7 ± 15.3	90 ± 20
	G63	7.5 ± 0.6	6.9 ± 0.4	6.0 ± 0.3	5.4 ± 0.5	5.0 ± 0.3	8.7 ± 0.8	8.7 ± 0.8	5.9 ± 1.5	11 ± 2
	G52	7.4 ± 0.7	6.9 ± 0.5	6.5 ± 0.3	5.9 ± 0.6	6.2 ± 0.4	6.5 ± 0.6	6.5 ± 0.6	7.9 ± 2.0	32 ± 8
	G53	9.1 ± 0.8	7.6 ± 0.5	7.7 ± 0.4	7.1 ± 0.7	7.1 ± 0.5	6.3 ± 0.5	6.2 ± 0.5	5.5 ± 1.5	8.6 ± 1.6
	T54	8.4 ± 0.7	9.1 ± 0.6	6.0 ± 0.3	5.0 ± 0.4	4.7 ± 0.3	6.0 ± 0.4	6.0 ± 0.4	55.1 ± 4.6	n. d.
	Ψ55-N3	9.1 ± 0.8	8.2 ± 0.5	7.7 ± 0.4	8.1 ± 0.7	9.3 ± 0.6	17.1 ± 1.5	19.2 ± 1.5	90 ± 20	90 ± 20
	Ψ55-N1	7.8 ± 0.7	12.6 ± 1.6	7.8 ± 0.4	8.5 ± 0.7	12.5 ± 1	70 ± 10	24 ± 2	n. d.	n. d.

Supplementary Table S 14: Determined water exchange rates (k_{ex}) of the imino protons in the non-modified tRNA^{fMet}. The k_{ex} rates are obtained by fitting the signal intensities of the imino resonances. 'n. d.' corresponds to 'not detectable'.

Nt.		k_{ex} [s^{-1}]								
		283 K	290 K	297 K	304 K	311 K	318 K	320 K	323 K	328 K
Acceptor stem	G2	9.2 ± 0.7	9.5 ± 0.7	6.2 ± 0.3	5.9 ± 0.3	8.2 ± 0.5	19.2 ± 2.5	10.7 ± 0.8	15.9 ± 1.9	24.5 ± 2.5
	G70	8.2 ± 0.6	8.3 ± 0.6	5.8 ± 0.3	5.3 ± 0.3	5.1 ± 0.3	6.7 ± 0.7	5.8 ± 0.4	6.6 ± 0.7	10.3 ± 0.9
	G4	10.5 ± 0.9	9.9 ± 0.8	6.8 ± 0.3	5.9 ± 0.3	5.1 ± 0.3	5.6 ± 0.6	5.5 ± 0.4	5.8 ± 0.6	7.7 ± 0.7
	G5	9.6 ± 0.7	10.4 ± 0.8	6.3 ± 0.3	5.4 ± 0.3	5.0 ± 0.3	5.2 ± 0.5	5.1 ± 0.3	6.1 ± 0.6	8.0 ± 0.7
	G6	9.3 ± 0.7	9.2 ± 0.7	6.1 ± 0.3	5.3 ± 0.3	5.6 ± 0.3	7.9 ± 0.8	6.5 ± 0.4	8.2 ± 0.8	16.1 ± 1.6
	G7	9.2 ± 0.7	9.5 ± 0.7	6.2 ± 0.3	5.9 ± 0.3	8.2 ± 0.5	19.2 ± 2.5	10.7 ± 0.8	15.9 ± 1.9	24.5 ± 2.5
D-arm	G15	n. d.	n. d.	n. d.	n. d.	n. d.	n. d.	n. d.	n. d.	n. d.
	G18	n. d.	n. d.	n. d.	n. d.	n. d.	n. d.	n. d.	n. d.	n. d.
	G22	10.5 ± 0.9	9.9 ± 0.7	6.8 ± 0.3	5.9 ± 0.3	5.1 ± 0.3	5.6 ± 0.6	5.5 ± 0.4	5.8 ± 0.6	7.7 ± 0.7
	G12	9.0 ± 0.7	8.9 ± 0.7	7.6 ± 0.4	8.7 ± 0.5	11.4 ± 0.7	12.4 ± 1.4	13.3 ± 1.0	12.6 ± 1.4	20.9 ± 2.2
	U24	n. d.	n. d.	n. d.	n. d.	n. d.	n. d.	n. d.	n. d.	n. d.
ACSL	U27	n. d.	n. d.	n. d.	n. d.	n. d.	n. d.	n. d.	n. d.	n. d.
	G29	8.5 ± 0.6	7.7 ± 0.5	6.5 ± 0.3	6.7 ± 0.3	9.8 ± 0.5	27.2 ± 3.7	11.3 ± 0.8	17.1 ± 2.1	23.7 ± 2.4
	G30	n. d.	n. d.	n. d.	n. d.	n. d.	n. d.	n. d.	n. d.	n. d.
	G31	9.3 ± 0.7	9.2 ± 0.7	6.1 ± 0.3	5.3 ± 0.3	5.6 ± 0.3	7.9 ± 0.8	6.5 ± 0.4	8.2 ± 0.8	16.1 ± 1.6
TΨC-arm	G49	9.2 ± 0.7	8.2 ± 0.6	5.8 ± 0.3	5.5 ± 0.3	7.5 ± 0.4	31.6 ± 4.6	10.5 ± 0.7	17.9 ± 2.2	22.8 ± 2.4
	U50	9.0 ± 0.7	9.3 ± 0.7	5.0 ± 0.2	4.8 ± 0.3	6.1 ± 0.3	19.1 ± 2.5	8.5 ± 0.5	15.0 ± 1.8	17.2 ± 1.7
	G64	9.7 ± 0.8	9.8 ± 0.8	8.9 ± 0.4	8.7 ± 0.5	11.0 ± 0.6	20.9 ± 2.5	11.9 ± 0.8	17.6 ± 1.9	44.4 ± 5.0
	G63	8.2 ± 0.6	8.3 ± 0.6	5.8 ± 0.3	5.3 ± 0.3	5.1 ± 0.3	6.7 ± 0.7	5.8 ± 0.4	6.6 ± 0.7	10.3 ± 0.9
	G52	8.9 ± 0.7	9.3 ± 0.7	5.4 ± 0.2	4.8 ± 0.3	6.1 ± 0.3	5.7 ± 0.6	8.5 ± 0.5	6.1 ± 0.6	9.3 ± 0.8
	G53	9.3 ± 0.7	9.2 ± 0.7	6.1 ± 0.3	5.3 ± 0.3	5.6 ± 0.3	3.6 ± 0.4	4.3 ± 0.3	3.9 ± 0.4	6.1 ± 0.4
	U54	9.6 ± 0.6	9.1 ± 0.6	6.8 ± 0.3	7.7 ± 0.4	20.2 ± 1.2	151.4 ± 19.8	30.4 ± 2.3	58.3 ± 8.5	97.5 ± 16.1
	U55	9.7 ± 0.8	9.8 ± 0.8	9.1 ± 0.4	14.1 ± 0.8	39.2 ± 2.8	18.2 ± 2.9	52.0 ± 4.7	29.4 ± 3.8	84.3 ± 21.1

Supplementary Table S15: Determined thermodynamic parameters of the base pair opening in the modified tRNA^{fMet}. The analysis was performed as described here. The temperature used in the calculation of $T\Delta S_{diss}$ and ΔG_{diss} was 25 °C. 'n. d.' corresponds to 'not detectable'. *ΔS could not be determined.

Nt.		ΔH_{diss} [kJ/mol]	$T\Delta S_{diss}$ [kJ/mol]	ΔG_{diss} [kJ/ml]
Acceptor stem	G2	-20 ± 30	-30 ± 30	20 ± 40
	G70	200 ± 70	170 ± 50	30 ± 90
	G4	60 ± 30	40 ± 30	20 ± 40
	G5	18 ± 18	-4 ± 16	20 ± 20
	G6	240 ± 60	200 ± 40	40 ± 70
	G7	50 ± 20	23 ± 19	20 ± 30
D-arm	G15	n. d.	n. d.	n. d.
	G18	n. d.	n. d.	n. d.
	G22	60 ± 30	40 ± 30	20 ± 40
	G12	50 ± 20	30 ± 20	20 ± 30
	U24	n. d.	n. d.	n. d.
ACSL	U27	160 ± 0	0 ± 0*	160 ± 0*
	G29	20 ± 30	10 ± 30	20 ± 50
	G30	130 ± 60	100 ± 50	30 ± 80
	G31	240 ± 60	200 ± 40	40 ± 70
TΨC-arm	G49	0 ± 20	-20 ± 20	20 ± 30
	U50	-20 ± 30	-30 ± 30	20 ± 40
	G64	210 ± 90	180 ± 60	30 ± 110
	G63	150 ± 70	120 ± 50	30 ± 90
	G52	180 ± 60	150 ± 40	30 ± 80
	G53	-20 ± 20	-33 ± 20	20 ± 30
	T54	400 ± 300	370 ± 130	100 ± 300
	Ψ55-N3	110 ± 30	90 ± 20	20 ± 40
	Ψ55-N1	60 ± 60	40 ± 50	20 ± 70

Supplementary Table S16: Determined thermodynamic parameters of the base pair opening in the non-modified tRNA^{fMet}. The temperature used in the calculation of $T\Delta S_{diss}$ and ΔG_{diss} was 25 °C. 'n. d.' corresponds to 'not detectable'.

Nt.		ΔH_{diss} [kJ/mol]	$T\Delta S_{diss}$ [kJ/mol]	ΔG_{diss} [kJ/ml]
Acceptor stem	G2	50 ± 20	40 ± 20	20 ± 30
	G70	60 ± 20	40 ± 20	20 ± 30
	G4	80.0 ± 30	60 ± 20	30 ± 40
	G5	90± 40	60 ± 30	30 ± 50
	G6	90 ± 50	70 ± 30	20 ± 60
	G7	50± 20	40 ± 20	20 ± 30
D-arm	G15	n. d.	n. d.	n. d.
	G18	n. d.	n. d.	n. d.
	G22	80 ± 30	60 ± 20	30 ± 40
	G12	16.3 ± 0.2	0 ± 0	16.3 ± 0.2
	U24	n. d.	n. d.	n. d.
ACSL	U27	n. d.	n. d.	n. d.
	G29	30 ± 20	11 ± 16	20 ± 20
	G30	n. d.	n. d.	n. d.
	G31	90 ± 50	70 ± 30	20 ± 60
TΨC-arm	G49	60 ± 30	40 ± 20	20 ± 30
	U50	60 ± 30	40 ± 30	20 ± 50
	G64	20 ± 30	0 ± 20	20 ± 30
	G63	60 ± 20	40 ± 20	20 ± 30
	G52	30 ± 40	10 ± 30	20 ± 50
	G53	140 ± 130	100 ± 90	30 ± 160
	U54	70 ± 30	50 ± 20	20 ± 40
	U55	20 ± 20	0 ± 20	20 ± 30

References

1. Berlyn, M. K. B. Linkage Map of Escherichia coli K-12, Edition 10: The Traditional Map. *Microbiol. Mol. Biol. Rev.* **62**, 1554 (1998).
2. Mandal, N. & RajBhandary, U. L. Escherichia coli B lacks one of the two initiator tRNA species present in E. coli K-12. *J. Bacteriol.* **174**, 7827–7830 (1992).
3. Kapoor, S., Das, G. & Varshney, U. Crucial contribution of the multiple copies of the initiator tRNA genes in the fidelity of tRNA fMet selection on the ribosomal P-site in Escherichia coli. doi:10.1093/nar/gkq760.
4. Goddard, T. D. *et al.* UCSF ChimeraX: Meeting modern challenges in visualization and analysis. *Protein Sci.* **27**, 14–25 (2018).
5. Pettersen, E. F. *et al.* UCSF ChimeraX: Structure visualization for researchers, educators, and developers. *Protein Sci.* **30**, 70–82 (2021).
6. Goddard, T. D. & Kneller, D. G. . SPARKY 3.
7. D', E. J., Ae, A., Gooley, P. R., D'auvergne, E. J. & Gooley, P. R. Optimisation of NMR dynamic models I. Minimisation algorithms and their performance within the model-free and Brownian rotational diffusion spaces. *J Biomol NMR* **40**, 107–119 (2008).
8. d'Auvergne, E. J. & Gooley, P. R. Optimisation of NMR dynamic models II. A new methodology for the dual optimisation of the model-free parameters and the Brownian rotational diffusion tensor. *J. Biomol. NMR* **40**, 121–133 (2008).
